# Synchrotron-Based X-ray Fluorescence Imaging Elucidates Uranium Toxicokinetics in *Daphnia magna*

**DOI:** 10.1021/acsnano.2c06111

**Published:** 2023-03-15

**Authors:** Ian Byrnes, Lisa Magdalena Rossbach, Dag Anders Brede, Daniel Grolimund, Dario Ferreira Sanchez, Gert Nuyts, Václav Čuba, Estela Reinoso-Maset, Brit Salbu, Koen Janssens, Deborah Oughton, Shane Scheibener, Hans-Christian Teien, Ole Christian Lind

**Affiliations:** † Centre for Environmental Radioactivity (CERAD), Faculty of Environmental Sciences and Natural Resource Management, Norwegian University of Life Sciences, P.O. Box 5003, 1433 Ås, Norway; ‡ Swiss Light Source, Paul Scherrer Institute (PSI), 5232 Villigen, Switzerland; § AXIS Group, NANOlab Center of Excellence, Department of Physics, University of Antwerp, Groenenborgerlaan 171, 2020 Antwerp, Belgium; ∥ Faculty of Nuclear Sciences and Physical Engineering, Czech Technical University in Prague, Brehova 7, 166 36 Prague 1, Czech Republic

**Keywords:** uranium nanoparticles, elemental distributions, X-ray absorption spectroscopy, tomography, synchrotron-based imaging, model organism, ecotoxicology

## Abstract

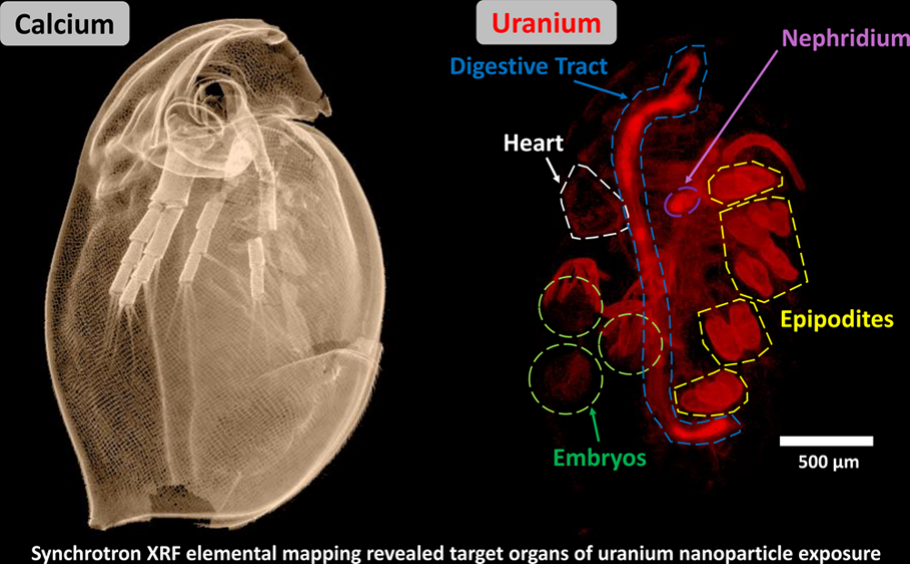

A combination of synchrotron-based elemental analysis and acute toxicity tests was used to investigate the biodistribution and adverse effects in *Daphnia magna* exposed to uranium nanoparticle (UNP, 3–5 nm) suspensions or to uranium reference (U_ref_) solutions. Speciation analysis revealed similar size distributions between exposures, and toxicity tests showed comparable acute effects (UNP LC_50_: 402 μg L^–1^ [336–484], U_ref_ LC_50_: 268 μg L^–1^ [229–315]). However, the uranium body burden was 3- to 5-fold greater in UNP-exposed daphnids, and analysis of survival as a function of body burden revealed a ∼5-fold higher specific toxicity from the U_ref_ exposure. High-resolution X-ray fluorescence elemental maps of intact, whole daphnids from sublethal, acute exposures of both treatments revealed high uranium accumulation onto the gills (epipodites) as well as within the hepatic ceca and the intestinal lumen. Uranium uptake into the hemolymph circulatory system was inferred from signals observed in organs such as the heart and the maxillary gland. The substantial uptake in the maxillary gland and the associated nephridium suggests that these organs play a role in uranium removal from the hemolymph and subsequent excretion. Uranium was also observed associated with the embryos and the remnants of the chorion, suggesting uptake in the offspring. The identification of target organs and tissues is of major importance to the understanding of uranium and UNP toxicity and exposure characterization that should ultimately contribute to reducing uncertainties in related environmental impact and risk assessments.

Uranium (U) is present in the environment due to releases from naturally occurring minerals^1^ or from anthropogenic sources such as those from the nuclear weapon and fuel cycles^2^ including global weapons fallout, U mining,^3,4^ and nuclear accidents (*e.g*., the Chernobyl accident),^5^ depleted U usage including military applications,^6^ and some non-nuclear related sources (*e.g*., the catalyst industry).^7^ Historically, assessments of U contamination in the environment have assumed a homogeneous distribution of ionic uranyl species of low molecular mass (LMM, <3 kDa) and have not accounted for the contribution of particles (>0.45 μm) and colloids (1 nm to 0.45 μm).^8^ Through weathering of larger particles or by direct precipitation as a result of biogeochemical processes, the prevalence of increasingly smaller particles gives rise to a log-normal size distribution that should be taken into account in environmental impact and risk assessments.^9−11^ Nanoparticles (NPs) have unique properties, such as a high surface-to-volume ratio, chemical reactivity, and high mobility, which may influence organism uptake and result in heterogeneous accumulation in tissues.^12^ As a result, there is a high degree of uncertainty about the long-term ecological consequences and risk posed by uranium NPs (UNPs) in the environment. Exposure to environmental U has a radiological and a chemical risk, of which the latter dominates (especially for natural U).^13^ Uranium concentrations in aquatic systems, such as freshwater ponds, frequently exceed the World Health Organization (WHO) guideline value of <30 μg L^–1^ in drinking water^14^ and can in some areas reach several mg L^–1^.^3,15^ Aquatic freshwater invertebrates, such as *Daphnia magna*, have a key function in nutrient cycling and constitute an essential part of the food web.^16,17^ In ecotoxicological assessments, *D. magna* is an important sentinel test organism with high sensitivity to metals, including U,^18−20^ but to date no toxicity studies have been published on UNP exposure. Traditional toxicity assessments have relied on measurements of total water concentration and whole body burden to provide overall uptake and depuration rates in *D. magna*. However, total water concentrations do not account for metal speciation in exposure media, and whole body burden measurements do not differentiate internal uptake from surface-bound or intestinally confined elements. Therefore, the customary methods of toxic assessment should be complemented with analyses that assess whole organism biodistribution and tissue-specific localization to better interpret subsequent effects.^21^

X-ray spectroscopic methods, including X-ray fluorescence (XRF) mapping, are powerful tools for investigating metals and metal NP distribution in biological samples.^22,23^ Despite the large body of toxicological research devoted to *D. magna*, specific metal uptake pathways and areas of tissue and organ accumulation remain largely unknown, with most work focusing on metallothionein expression studies or XRF imaging of the intestine.^24−28^ Furthermore, U biodistribution in daphnids remains mostly unexplored, as research has been primarily focused on cell level histological analyses.^29^ Recent advances in analytical synchrotron technology enable assessment of tissue-specific trace metal distributions that are highly useful for examining potential uptake pathways and tissue accumulation in daphnids.^30,31^ Applying such techniques to ecotoxicological studies could provide valuable insights into the overall toxic assessment and fill knowledge gaps with respect to toxicokinetics and toxic mode of action, required for aggregate exposure pathway (AEP) framework development.^32,33^

Therefore, this study investigated the accumulation and distribution of U in *D. magna* following acute exposure to aqueous UNPs or U reference solutions (U_ref_) by utilizing highly sensitive, microfocused XRF elemental mapping and XRF tomography to determine the tissue level localization in preserved intact organisms. This approach provided detailed data on U uptake and accumulation, retention in target organs, and detoxification pathways, thereby providing important information that links exposure and biodistribution to toxic effects.

## Results and Discussion

### Uranium Nanoparticle and Exposure Characterization

The X-ray diffraction (XRD) analysis showed that the engineered, dry UNPs consisted predominantly of uranium dioxide (UO_2_, Figure S1), although the subsequent micro-X-ray absorption near-edge structure (μ-XANES) analysis indicated that oxidation of the UNPs (*i.e*., from U(IV) to U(VI)) had occurred since the time of synthesis (Figure S2). According to transmission electron microscopy (TEM) analysis of the UNP stock suspension, individual NPs featured physical diameters of 3–5 nm (Figure S3). The average hydrodynamic diameter of the UNP stock was 273.3 ± 1.2 nm (Table S1) with a zeta potential of −11.8 mV, indicating an unstable suspension, where Coulomb repulsive forces were not sufficient to prevent further aggregation.^34^ Elemental composition analysis, measured by triple quadrupole inductively coupled plasma mass spectrometry (ICP-QQQ), showed the presence of several trace elements (*i.e*., B, Ti, Mo, V, Ag, and Sn) in addition to U (Table S2). All trace metals remained below reported toxic effect levels for *D. magna*.

Experiments were carried out in moderately hard reconstituted water (MHRW, pH 6.8), and size fractionation analyses of the exposure media showed similar LMM (<3 kDa), colloidal (3 kDa < *x* < 0.45 μm), and particulate (>0.45 μm) size distributions for both the UNP and U_ref_ exposures (Figure S4). In general, the colloidal and particulate fractions (>3 kDa) comprised between ∼50% and 80% of total U in either exposure treatments. The formation of colloids and particulates was expected in the UNP exposure media due to aggregation. The ∼20% to 50% LMM fraction in the UNP exposure media also signified substantial particle dissolution. Since μ-XANES of dry powders indicated oxidation of the UNPs, dissolution and concomitant formation of LMM species in the MHRW solution are plausible.

Follow-up analysis of the U_ref_ media, via TEM, identified U-bearing, nanoscale (5–10 nm) crystalline structures that may constitute the colloidal and particulate fraction in those exposures (Figure S5). The aqueous speciation of U is complex due to the formation of a variety of hydrolysis products and complexes with inorganic and organic ligands.^35^ In exposures of >200 μg U L^–1^ at pH 6.8 in MHRW-comparable media, the U speciation would include (UO_2_)_2_(OH)_2_CO_3_^–^, UO_2_CO_3_, UO_2_(OH)_2_, and UO_2_OH^+^, as well as dimeric and polymeric U species.^36,37^ This behavior potentially influences both uptake and toxicity of both UNPs and U_ref_ species.

### Determination of Toxic Effects

The acute toxicity tests in the current study were designed to remain within the tolerable pH range for *D. magna*,^16^ while maximizing the bioavailable U fraction. Therefore, MHRW^38^ adjusted to pH 6.8 was chosen as exposure media to minimize the presence of uranyl-complexing ligands as much as feasible. Standard acute toxicity tests were conducted with both neonates (<18 h) and adults (<7 d) to assess mortality and total body burden. In line with the speciation analysis (Figure S4), the results revealed a similar dose–response relationship for mortality as a function of the measured total U water concentration from both UNP and U_ref_ exposures (Figure S6). Furthermore, significant effects (ANOVA, *p* < 0.05) were observed at concentrations ≥388 ± 10 μg U L^–1^ and ≥260 ± 13 μg U L^–1^, for UNP and U_ref_ exposures, respectively. The calculated lethal concentration in 50% and 10% of the population (LC_50_ and LC_10_ values, Table S3) revealed differences in acute effects (*e.g*., mortality) between exposures to the U_ref_ (LC_50_ of 268 μg U L^–1^ [229–315]) and the UNPs (LC_50_ of 402 μg U L^–1^ [336–484]), although overlap in the respective LC_10_ 95% credible limits of 130–238 μg U L^–1^ for UNPs and 97.8–168 μg U L^–1^ for U_ref_ exposures was observed. The neonates were approximately 4-fold more susceptible than the adults with an LC_50_ of 127 μg U L^–1^ [102–163] for the UNP suspension and an LC_50_ of 112 μg U L^–1^ [89.5–136] for the U_ref_ solutions. Furthermore, the predicted LC_10_ 95% credible intervals were calculated to be 35.7–73.4 μg U L^–1^ and 26.5–62.0 μg U L^–1^ for UNP and U_ref_, respectively. Acute toxicity of U is dependent on several physicochemical parameters of the exposure media and is closely connected to the U aqueous speciation, indicated by the large LC_50_ concentration range for U in *D. magna* in previous studies (0.39–6.4 mg U L^–1^).^18,19,39^ The observed LC_50_ values were slightly lower than those previously reported for dissolved U at similar pH levels,^13,18,39^ which could be related to the U speciation in the media solution, where bioavailability is closely linked to the pH-dependent abundance of uranyl ions, such as UO_2_^2+^ and UO_2_OH^+^.^40^

The total body burden following exposure to the UNPs and U_ref_ exposures was investigated to evaluate potential relationships between U aqueous concentrations, accumulation, and survival. The results revealed that the U body burden (ng U daphnid^–1^) correlated to the total water concentration for both treatments (Figure S7). The U body burden in neonates showed similar concentrations for UNPs (0.7 ± 0.5 to 3.2 ± 0.2 ng U daphnid^–1^) and the U_ref_ (0.8 ± 0.5 to 5.6 ± 1.9 ng U daphnid^–1^). However, total body burden for adults exposed to UNPs (8.5 ± 3.3 to 64.6 ± 22.0 ng U daphnid^–1^) was approximately 3-fold higher than those exposed to the U_ref_ (6.5 ± 0.7 to 20.3 ± 0.4 ng U daphnid^–1^). These results suggest that, although the size distribution (Figure S4) was similar between the exposures, functional differences between U species related to uptake and bioavailability led to the higher body burden of UNPs in adult daphnids compared to the U_ref_. The UNPs, and aggregates thereof, were likely captured more effectively than the U_ref_ species by the daphnid filter feeding apparatus or by feed material remaining in the intestine of the adult daphnids (neonates never received feed). Therefore, survival was plotted as a function of body burden to evaluate potential differences in specific toxicity (Figure S8). Regression analysis showed a statistically significant linear correlation (*R*^2^ = 0.53 for U_ref_ and 0.63 for UNP, *p* < 0.05) between body burden and survival of adult daphnids. Furthermore, the slope of the regression curve was 6-fold steeper for U_ref_ compared to UNP (Figure S8). The observed narrow range from no effect at <10 ng U daphnid^–1^ to 90% mortality in 20 ng U daphnid^–1^ implied the presence of U species with high specific toxicity in the U_ref_ exposure, whereas a high proportion of less toxic species were present in the UNP exposures, as indicated by the 3- to 5-fold higher body burden. However, uptake, accumulation, and excretory pathways of U in daphnids remained unclear. Therefore, synchrotron X-ray analyses were used to assess the U biodistribution in organisms exposed to sublethal concentrations of UNPs and the U_ref_.

### Uranium Biodistribution

**Figure 1:**
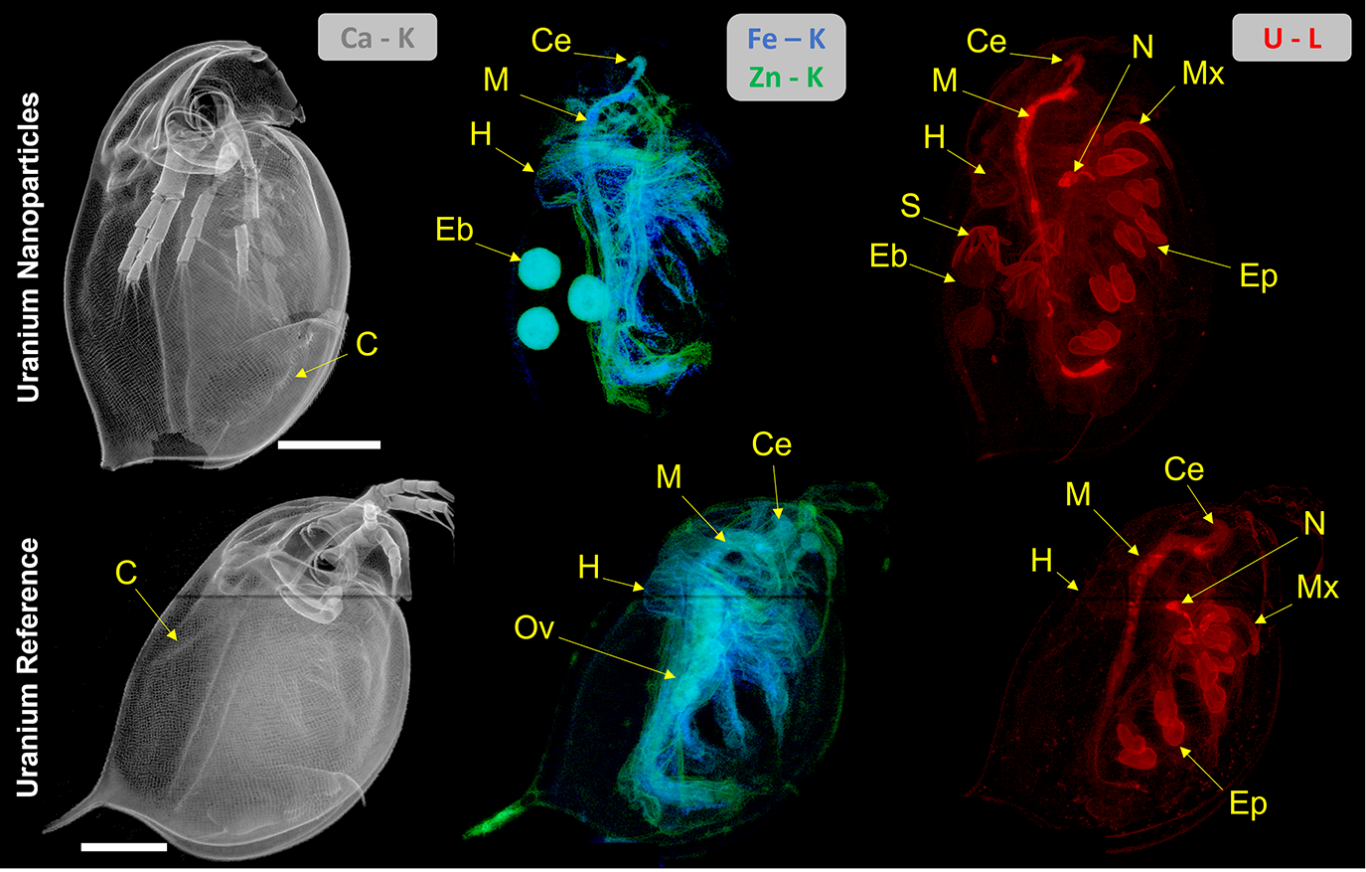
Whole body XRF elemental maps of *D. magna* exposed to either 320 μg U L^–1^ UNP (upper panel) or 159 μg U L^–1^ U_ref_ (lower panel). Calcium distribution was indicative of the carapace, while Fe and Zn indicated soft tissues (displaying fitted K fluorescent peaks). The U (fitted L fluorescent peaks) biodistribution is shown on the right. Both scans were conducted with a 5 μm step size and 200 ms dwell time. All scale bars represent 500 μm, and all elemental signal intensities are scaled logarithmically (linear scale maps with intensity scale can be found in Figure S10). Abbreviations: carapace (C), maxillary gland (Mx), nephridium (N), epipodites (Ep), chorion structures (S), hepatic ceca (Ce), midgut (M), heart (H), embryo (Eb), and ovary (Ov).

Intact, adult *D. magna* individuals (Figure S9), exposed to either 320 μg U L^–1^ UNP or 159 μg U L^–1^ U_ref_ followed by rinsing in clean water, were prepared and imaged using synchrotron-based XRF elemental mapping at 5 μm resolution to obtain whole body biodistributions of U and essential elements such as Fe and Zn, indicative of soft tissues, as well as Ca, which is associated with the chitinous carapace (Figure 1). Uranium was distributed throughout most tissues of the daphnid (*n* = 1) from both the UNP and the U_ref_ exposures with comparable areas of accumulation. Areas of significant U accumulation included the gills (epipodites), inside the digestive tract, and the maxillary gland (Figure 1). Additionally, U was also present on the carapace surface, within soft tissues including the heart, and within the brood chamber and embryos, albeit at lower intensities. It is worth noting that although the accumulation areas were comparable between the two treatments, the U_ref_-exposed daphnid featured higher U signal intensities on the epipodites compared with the UNP-exposed organism (Figure S10). Additionally, despite the use of a single, synchronized cohort in the exposures, the studied U_ref_ specimen had not yet undergone oviposition at the time of sampling; thus no embryos could be observed in the brood chamber of that specimen.

These observations are in line with previous assessments of U toxicity in *D. magna* that indirectly reported accumulation in the intestine via observation of histological changes to epithelial cells^29^ and on the carapace, as well as maternal transfer.^20^ However, the biodistribution in the current study provides much more detailed information on whole body uptake and distributions. The following sections describe the identified areas of U accumulation of importance to toxicokinetic and toxicodynamic assessments.

#### Surface-Bound Uranium

Based on whole body XRF scans (Figure 1), surface-bound U was illustrated by a low signal coinciding with the Ca-rich carapace and a high accumulation on the epipodites, which are located on the end of each thoracic appendage. Some U may have been removed from the surface of the daphnid during the sample preparation, as indicated by <10% U total loss measured in the solutions used in the low-impact chemical drying procedure^42^ (Figure S11). The observation of the U surface distribution has important implications for understanding uptake and depuration pathways for *D. magna*. Accumulation on the epipodites may reflect a potential uptake pathway via ion exchange with the surrounding media.^43,45^ On the other hand, removal of excess U from the carapace and epipodites via molting represents a significant depuration pathway for daphnids.^20,43^

**Figure 2:**
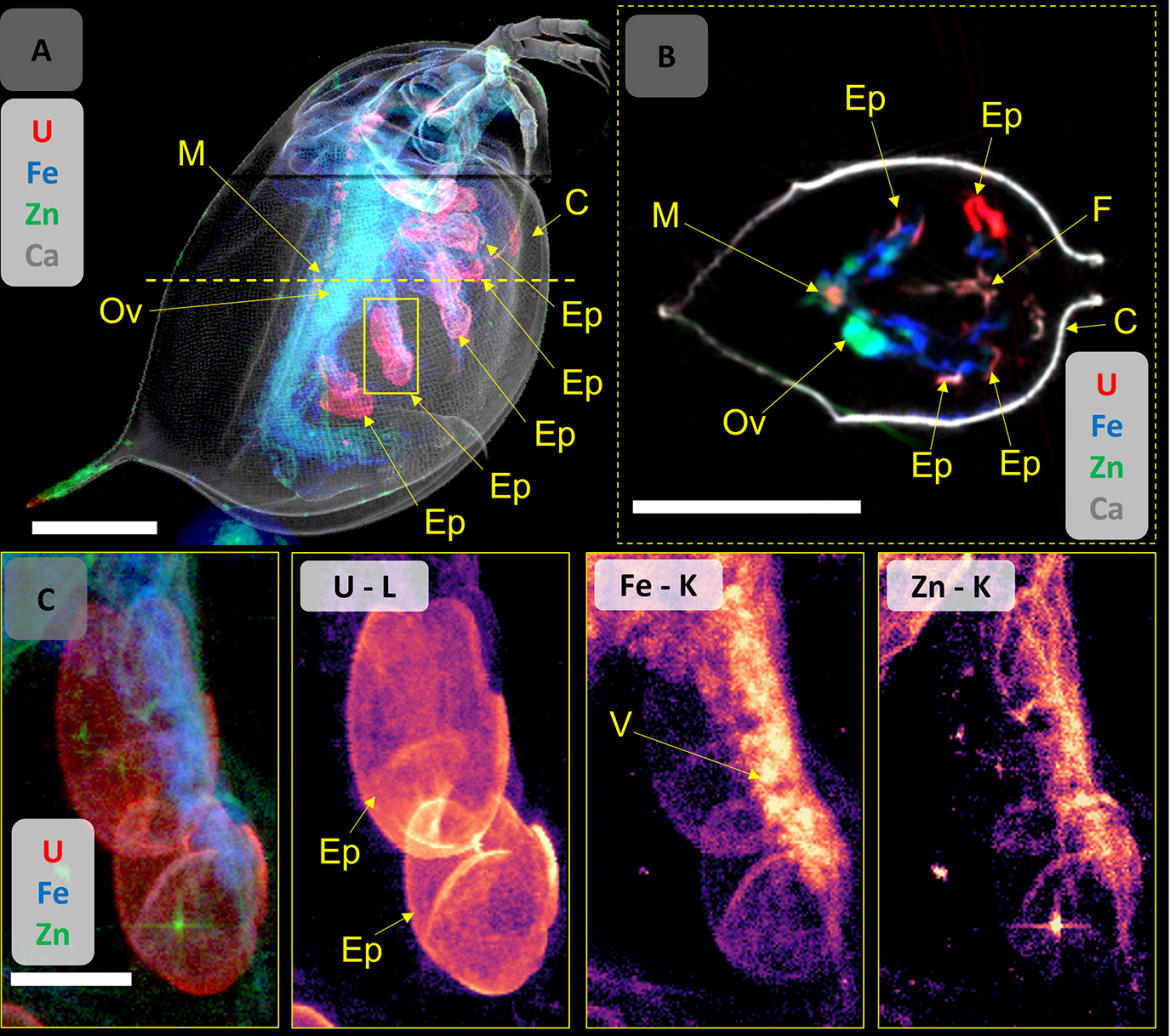
(A) Composite elemental map (U, Fe, Zn, Ca) of *D*. *magna* exposed to the U_ref_ (159 μg U L^–1^), indicating accumulation of U onto the epipodites, the area chosen for 2 μm high-resolution mapping (yellow box), and the location of the tomographic section (yellow dotted line). (B) Tomographic section (2 μm resolution) showing the dorsal distributions of U, Fe, Zn, and Ca. The U distribution map in intensity scale can be found in Figure S14. (C) High-resolution, two-dimensional projections of the epipodites showing U, Fe, and Zn distributions (composite and individual maps). Note: the star-shaped signal in the Zn map is an artifact, likely an external contamination of the sample (*e*.*g*., dust particles) and should not be interpreted as part of the daphnid. Scale bars represent 500 μm (A), 50 μm (B), or 100 μm (C), and all elemental signal intensities are scaled logarithmically. Abbreviations: epipodite (Ep), carapace (C), midgut (M), vesicle (V), ovary (Ov), and food groove (F). Hotspots in the Zn elemental map were due to dust contamination on the surface of the sample.

High-resolution (2 μm) region of interest (ROI) scans of the epipodites provided a readily identifiable representation of the U accumulation in these ∼100 μm sized organs, shown clearest in the U_ref_-exposed organism (Figure 2). In these 2D XRF projections, the U appeared concentrated on the organs compared with other surrounding soft tissues, which exhibited a much lower U intensity (Figure 2A,C). The high-intensity U signal associated with the epipodites obscures U associated with structures behind them that may be the Fe-rich vesicle running into the organ. High-resolution maps of the epipodites (Figure 2C) showed the Fe-rich vesicle extending through the appendage, further supporting previous assertions that such tissues are responsible for hemoglobin synthesis and connected to the circulatory system.^43,45^ Comparing the measured intensity of U accumulated on the epipodites of the two U treatments, the U_ref_-exposed organism showed a ∼20% higher signal (counts per second) than the UNP-treated daphnid (Figure S12). Uranium intensity on the epipodites may reflect the amount of bioavailable U species present in the exposure; however, further analysis is needed to confirm such an assumption.

Using XRF tomographic sections, U appears to be associated with the ∼0.5 μm cuticular and ∼20 μm epithelial layers^43,45^ of the epipodites (Figure 2B), but not within the interior hemolymph space of the organ, which may have been evacuated by the dehydration process. The U distribution in the epipodites may resemble that observed in nano-XRF mapping of histological sections of Zn-exposed *D. magna*,^44^ where Zn was observed mainly on the surface, cuticular layer of the epipodite. Although the tomographic sections presented here suggest U is distributed evenly through the epithelial layer, finer resolution is needed to properly resolve the cuticular layer from the epithelial cells. Signal analysis of the U_ref_ organism’s epipodite (Figure S13) shows statistically significant correlation of U with Zn and Fe (Spearman’s *r*_s_ > 0.73 and 0.72, respectively). Similar correlations were also observed in the UNP-exposed organism (Figure S14). Finally, as seen in the 2D projection maps, the epipodite derived from the U_ref_-exposed daphnid in the tomographic section showed a higher U intensity than the counterpart from the UNP-exposed organism (Figure S15).

A previous toxicokinetic assessment showed that molting (ecdysis) is an important depuration pathway for *D. magna* exposed to U,^20^ and the biodistribution mapping in the current study provided further insights and detail to specific areas where U is lost. For *D. magna*, molting is not limited to the general carapace but also includes the cuticle surrounding the epipodites and the intestinal foregut and hindgut.^43,48^ Therefore, findings from the current study suggest that a major contribution to U depuration via molting may be attributed to shedding of epipodite cuticular layers and the fore- and hindgut, rather than from the general carapace surface.

#### Ingestion

**Figure 3:**
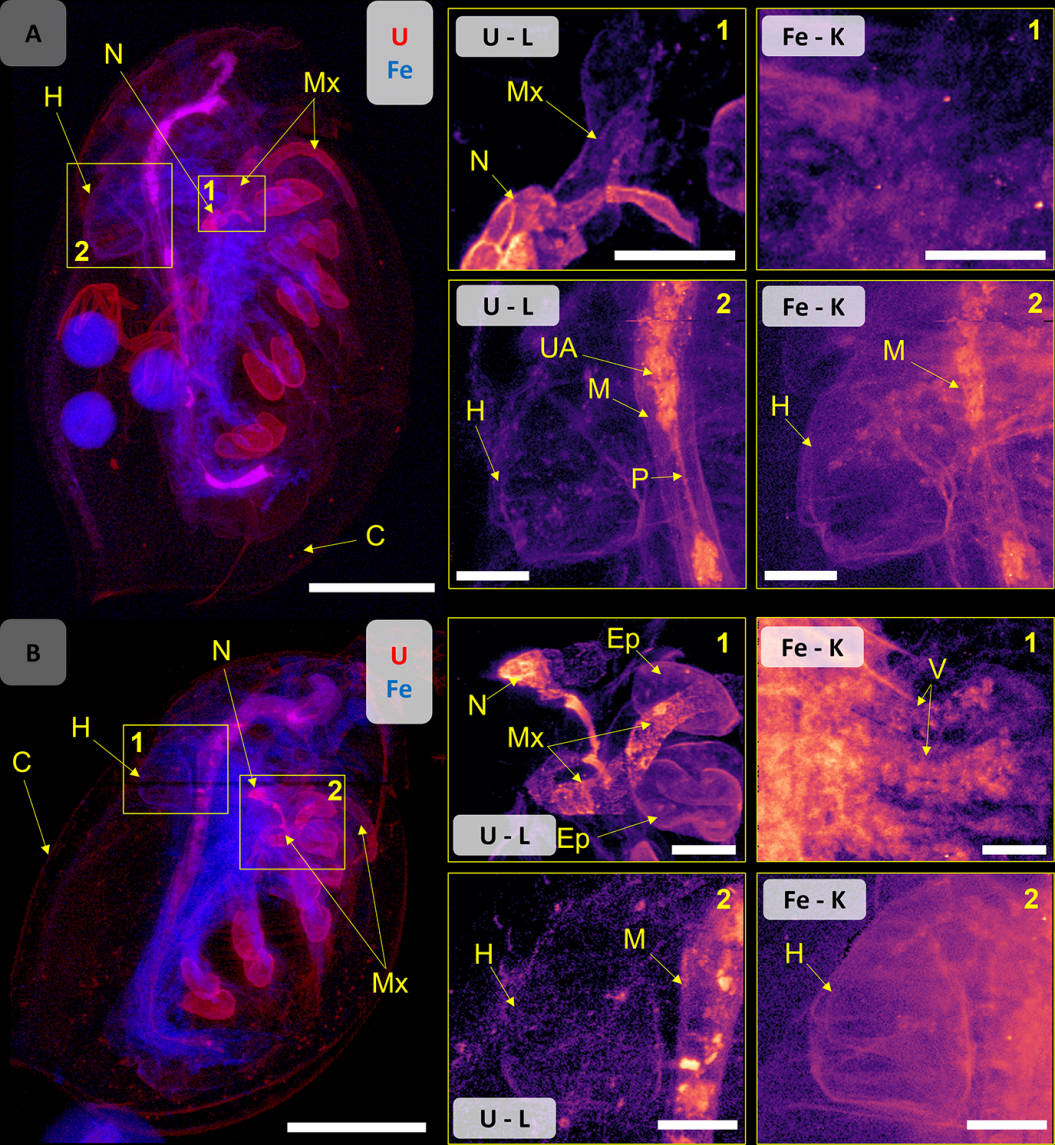
Composite map of U (red) and Fe (blue) of (A) UNP-exposed *D. magna* (320 μg U L^–1^) and (B) U_ref_-exposed organism (159 μg U L^–1^). High-resolution (2 μm) ROI maps showing the maxillary gland and nephridium (region 1) and the heart (region 2). Scale bars represent 500 μm on the whole daphnid maps and 100 μm on the ROI maps. Elemental signal intensities are in logarithmic scale. Abbreviations: vesicle (V), maxillary gland (Mx), nephridium (N), epipodite (Ep), UNP aggregates (UA), midgut (M), peritrophic membrane (P), heart (H), and carapace (C).

*D. magna* from both exposures (UNP and U_ref_) exhibited significant amounts of U particulates in the midgut region, an area important for digestion and nutrient uptake (Figure 3). Previous studies suggest that the midgut function is vulnerable to U exposure, and failure to properly assimilate nutrients may constitute a major toxic effect.^29,39^ In the current study, U-containing materials in the digestive tract were observed in daphnid derived from both exposures (Figure 1, Figure 3). Despite removing the daphnid from feed 24 h prior to the experiments, the U-bearing materials may include partially digested algae,^40^ which is consistent with previous findings showing that green algae species have the capacity to effectively bind U.^41^ Detailed mapping of the UNP-exposed daphnid intestines showed small, very high U intensity hotspots, which were a single pixel in size, likely corresponding to NP aggregates in the midgut (Figure 3A). These UNP aggregates may have been ingested or formed through aggregation promoted by the daphnia gut chemistry, as seen for other types of NPs.^49^ Additionally, the filter feeding behavior of daphnids^46^ may have contributed to the ingestion of particle aggregates from the media, which could also explain the high body burden in the UNP-exposed individuals observed in the current study (Figure S7). Importantly, U was found in the soft tissue structures of the midgut of all exposed organisms, which is a strong indication that the intestine constitutes an important uptake pathway (Figure 3A,B).

#### Systemic Uptake

The XRF imaging results of UNP- and U_ref_-exposed daphnids suggested two potential U uptake pathways into the hemolymph circulatory system, *i.e*., via the epipodite gill tissues and by translocation across the intestinal barrier. In the current study, the dehydration and drying of sample organisms for imaging precluded the assessment of the hemolymph; therefore, systemic uptake was sought after by assessing U in muscle tissues and internal organs (Figure 3). The fact that U was detected in the heart, albeit at low relative intensity compared to other organs and tissues, is unequivocal evidence of systemic uptake into the circulatory system. Furthermore, high U intensities observed in the maxillary gland and nephridium (Figure 3) corroborated systemic uptake. The maxilliary gland and nephridium represent a kidney-like organ system proposed to participate in osmoregulation and excretion of metabolic byproducts.^47^ These observations are consistent with the nephrotoxic mode of action of U.^37,50^ The elevated U levels in the maxillary gland and nephridium thus implies that the removal of U from the hemolymph via this organ system may represent a hitherto unidentified metal detoxification pathway in *D. magna*. Unfortunately, the maxillary gland and nephridium were not clearly visible in other elemental maps or in the unexposed control organism (Figure S16), probably due to the structure and composition primarily consisting of elements that were either below detection limits or not detectable by XRF, such as carbon. Additionally, this region of the organism also contains large soft tissue structures associated with the appendages, which may be denser and obscure the signal of essential elements, such as Zn, in 2D projection mapping. Since the maxillary gland is involved in excretion of ferrous breakdown products,^47^ it is thus conceivable that U may follow a pathway from uptake via the epipodites and/or the intestine, into the circulatory system and excretion via the nephridium and maxillary gland. Previously published depuration rates for U in *D. magna* reflected a 75% loss after 24 h, out of which 50% was bound to the carapace and shed by molting.^20^ Therefore, the remaining 25% might represent a combination of egestion of intestinal content and excretion through the maxillary gland. The latter may serve as an important function for U removal from the hemolymph and thus reduce toxicity to internal cells, tissues, and organs.

#### Uranium in the Brood Chamber

**Figure 4:**
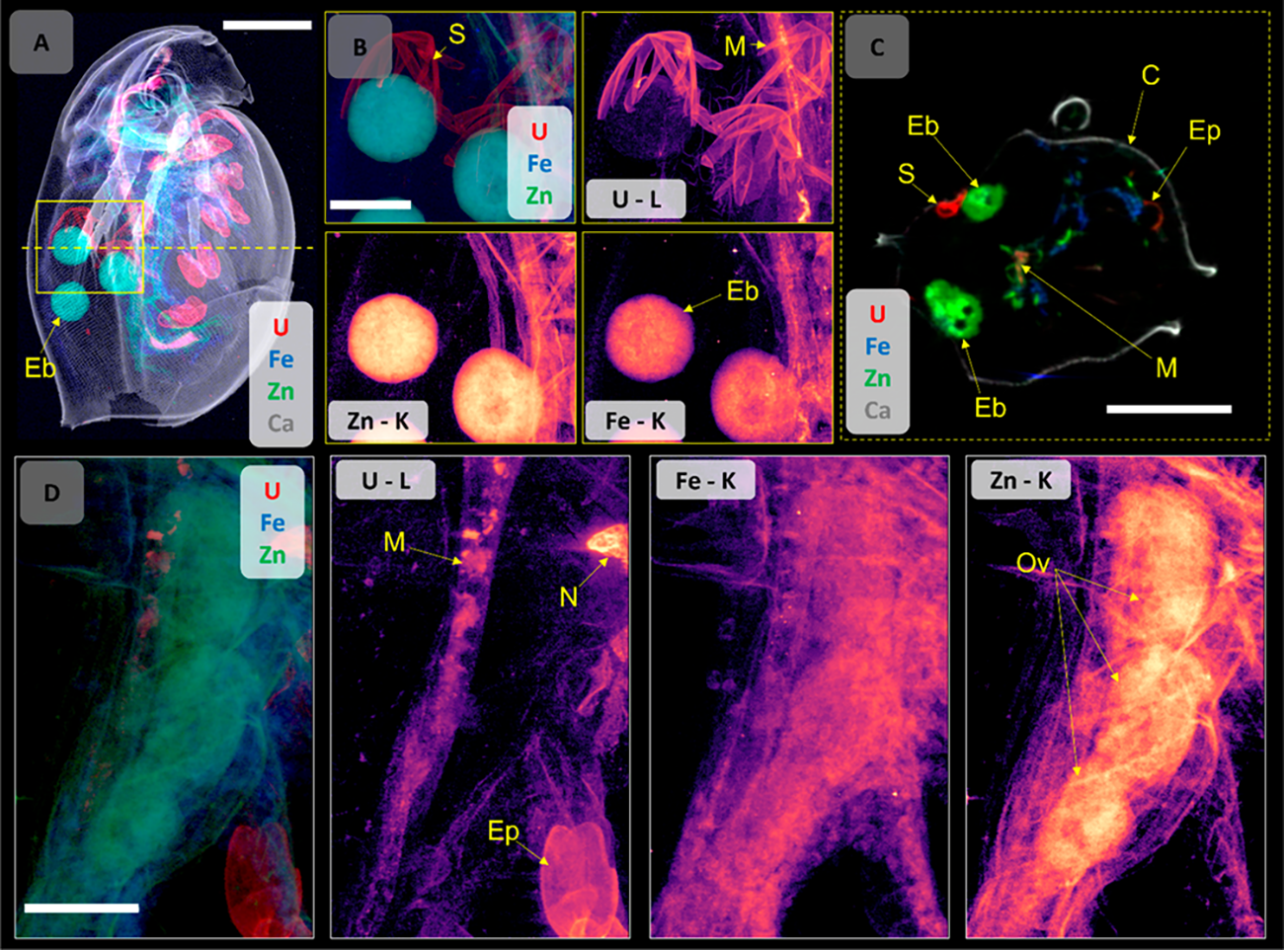
(A) Overview, combined elemental map (5 μm step size) of a UNP (320 μg U L^–1^)-exposed *D*. *magna* for U, Ca, Fe, and Zn distributions, indicating the ROI area chosen for 2 μm high-resolution mapping (yellow box) and the location of the tomographic section (yellow dotted line). (B) Elemental distribution (combined and individual maps) on the ROI showing the U-bearing chorion structures and embryos in the brood chamber. The U distribution map in intensity scale can be found in Figures S14 and S15). (C) A tomographic section (2 μm resolution) showing the distributions of U, Fe, Zn, and Ca in the brood chamber, including the U-bearing structures and embryos. (D) High-resolution elemental distribution maps (combined and individual) for U, Fe, and Zn of a ROI on the ovary of a U_ref_-exposed *D. magna* (159 μg U L^–1^, see Figure 2A for whole organism), where U is not observed in the developing embryo. Elemental signal intensities are in logarithmic scale. Scale bars represent 500 μm (A, C) and 200 μm (B, D). Abbreviations: embryo (Eb), chorion structures (S), midgut (M), epipodite (Ep), carapace (C), nephridium (N), and ovary (Ov).

Based on elemental mapping of the brood chamber of the UNP-exposed daphnid, U was detected in embryos and chorion structures inside the chamber (Figure 4, Figure S17). Such findings may have implications for the potential development of offspring and the long-term stability of a population. The observation of U in the embryos may be the result of direct exposure or via maternal transfer. The brood chamber of *D. magna* remains open to the outside environment,^55^ but the interior fluid is to some extent regulated by the parent, as evidenced by an increase in Na^+^ and Ca^2+^, to support embryo development.^56^ Therefore, the U seen in embryos in the brood chamber of the UNP-exposed daphnid shown in Figure 4 could have occurred directly via the interior fluids.

Alternatively, U taken up into the organism could be transferred maternally from parent to offspring, which in previous studies comprised approximately 1–7% of total body U in exposed animals,^20,51^ while studies of multigenerational U exposure also indicated long-term population effects.^29,52^ Interestingly, in the current study, no U signal was detected in three oocytes present in the ovary of the U_ref_-exposed animal, which had not yet undergone oviposition (Figure 4D). Such observations lend support to the previous notion that U observed in the embryos was derived from direct exposure in the brood chamber, rather than maternal transfer, or that maternal transfer occurs very late in the process of oviposition. Studies have shown that hemoglobin is produced in epipodite adipocyte tissues^45^ and subsequently transferred via lipid droplets into developing oocytes inside the ovary in the final stages prior to oviposition.^53^ Recently, maternal transfer of Ag^+^ and Ag NP was documented via these lipid droplets,^54^ and it is conceivable that maternal transfer of U may be facilitated by a similar process; however, further work is needed to investigate these mechanisms.

Within the brood chamber and around the embryos, the UNP-exposed organism exhibited U-containing structures that appeared to be the remains of a protective outer layer of the embryo (Figure 4A–C). To protect the developing embryo from any hostile environmental conditions, *D. magna* eggs develop a double-layered envelope with chorion immediately after oviposition.^53,57,58^ These structures did not appear in other elemental maps, indicating that the constituents were less than the LOD or not detectable by XRF, such as carbon. Additionally, XRF mapping of the control organisms did not show these structures, suggesting that the fractured chorion was a result of the U exposures (Figure S16). The chorion has been shown to accumulate hazardous materials including Ag^+^, Ag NPs, and polystyrene beads,^54,59^ and it is probable that U is transferred in a similar manner.

Studies of chronic U toxicity (21 d) in *D. magna* suggest effects on reproduction (reduced fecundity in first brood) starting at concentrations of 25 μg U L^–1^ (pH 7).^39^ Chronic exposure to U has also been shown to induce a reduction of body size and fecundity in subsequent generations of daphnids.^52^ In the present study, daphnids derived from exposures below the LC_50_ maintained in clean media and feed were able to reproduce despite U uptake during the embryonal stages (Table S4). The results in the present study provided visual evidence of U uptake into *D. magna* offspring, demonstrating that the egg envelope was unable to prevent U from entering embryos, which potentially may cause effects on subsequent generations.

## Conclusions

The current study employed state-of-the-art integrated methods to link the U uptake and biodistribution to toxic effects in *D. magna* following exposure to UNPs and U_ref_ solutions. Whole body XRF elemental mapping combined with detailed exposure characterization and toxic effects analysis provided insights into U accumulation and associated toxicokinetics. The results contribute to an improved understanding with respect to routes of U uptake, tissue and organ accumulation, potential transfer to or contamination of embryos, and organism detoxification. Furthermore, this study demonstrates the utility of synchrotron-based X-ray techniques in identifying target organs of exposure, a method that could be applied to other similar sized organisms and which is critical for construction of an AEP framework to guide toxicokinetic research. The identification of high U accumulation in target organs and tissues is scope for future investigation, such as the impacts on intestinal function and the surrounding soft tissues as well as the role of the epipodites and maxillary gland on the uptake and excretion of U and other toxic metals.

## Experimental Section

### Uranium Nanoparticle Synthesis and Characterization

Engineered UNPs were produced from uranyl nitrate (UO_2_(NO_3_)_2_) at the Czech Technical University. The synthesis procedure is described in detail in the Supporting Information, Section S1. The UNPs were stored as dry powders in Eppendorf tubes inside a desiccator at room temperature (20 °C) and atmospheric pressure until use.^60,61^ Dry powders were characterized by laboratory-based XRD and synchrotron-based μ-XANES analysis (details in synchrotron analyses section). For exposure experiments, UNP stock suspensions were prepared in ultrapure water. Average hydrodynamic diameter and zeta potential of stock suspensions (1.0 g U L^–1^) were characterized using dynamic light scattering (DLS), individual particle size was determined by TEM analysis, and fractionation experiments were conducted on selected concentrations from the UNP and U_ref_ exposures to determine the U species size distribution. A detailed description of these techniques and methods can be found in Section S2 of the Supporting Information.

### *Daphnia magna* Exposure and Sample Preparation

Laboratory *D. magna* cultures (DHI Water & Environment, Hørsholm, Denmark) were used in exposures according to a standard acute (48 h) toxic elemental test protocol^62^ without feed (Supporting Information, Section S3). All *D. magna* exposures were conducted using a range of UNP concentrations from 0– to 781 ± 85 μg U L^–1^ in MHRW (pH 6.8)^36^ at 20 °C with at least 20 animals in groups of 5, in accordance with the test protocol.^62^ The UNP exposures were compared with a similar concentration range (0 to 790 ± 42 μg U L^–1^) of a U_ref_ solution that was prepared from an acid U standard solution (1.0 g L^–1^ in 2% HNO_3_; CRM 129-A, U.S. Department of Energy, Argonne, IL, USA). According to the toxicity test protocol, daphnid mortality was considered an immobilized individual that was not able to swim for 15 s following gentle agitation.^62^ In all tests, no immobilized individuals were observed in the control group, meeting the validity requirement of the test protocol (<10% immobilized in control groups). Acute toxicity concentrations, 48 h LC_50_ and LC_10_, were determined with the MOSAIC tool for ecotoxicology assessments.^63^ MOSAIC employs a Bayesian model in the R package “morse”, which uses observed survival at each exposure concentration as inputs.^64^ One-way ANOVA was applied to assess for simple group comparison when residuals were normally distributed, using MinitabVR 18 (Minitab Inc. 2010). Where data were nonparametric, the Kruskal–Wallis test was employed.

Following the acute exposure, daphnids (F0) from sublethal concentrations were transferred to clean MHRW and maintained with feed for 24 h to observe reproduction (Supporting Information, Section S3). Additionally, an acute toxicity test was conducted using *D. magna* neonates (<18 h) to better compare results with literature LC_50_ values. All reported concentrations and LC_50_ values refer to measured concentrations in the exposure media.

After determining the LC_50_ for both exposures, adult daphnids (<7 d) were exposed to sublethal U concentrations (320 ± 31 μg U L^–1^ UNP, 159 ± 14 μg U L^–1^ U_ref_, and a control) for 48 h in MHRW (pH 6.8, 5 mL per daphnid). Microfocused, XRF, and X-ray absorption spectroscopy (XAS) measurements were conducted on whole, intact organisms (*n* = 1) that were preserved by chemical drying. In brief, daphnids were rinsed once with MHRW and twice with deionized water and then fixed in 5% methanol for 10 min.^65^ Subsequently, samples were dehydrated following a stepwise protocol of graded acetone (*i.e*., once with 70%, 80%, and 90% for 10 min each and twice with 98% and 100% for 10 min each). Lastly, samples were immersed in 2 mL of hexamethyldisilazane (HMDS) for 1 h.^39^ After removal of approximately 90% HMDS, samples were dried overnight in a desiccator with an applied vacuum of 200 mbar. Dried samples were gently moved into new Eppendorf tubes avoiding external contamination and kept at room temperature until analysis.

### Synchrotron-Based X-ray Analyses

Elemental distribution mapping of whole *D. magna* was conducted at the microXAS beamline (X05LA) at the Swiss Light Source (Paul Scherrer Institute, SLS, Switzerland). Daphnid samples were mounted on Kapton tape or glued to the fine tip of a wooden toothpick (Figure S9). A 17.2 keV incident beam was microfocused using a Kirkpatrick-Baez (KB) mirror system to a size of 1 μm^2^, and samples were raster-scanned in projection mode with a step size of 5 μm for whole organism scans and 2 μm for ROI maps. A photon flux of 2 × 10^10^ ph s^–1^ was obtained. Based on the resulting 2D projection map, XRF virtual slices were collected with computed tomography analysis, by collecting line profile projections at different orientations over 180° in 1° increments. All XRF spectra were collected using four silicon drift detectors (SDD; Ketek GmbH, Germany) positioned around the sample at 50° to the incoming beam, at approximately 2 cm from the samples with a 200 ms dwell time. The computed tomography data set was reconstructed using microXAS homemade python scripts using the ASTRA Toolbox library (FBP and SIRT),^66,67^ whereas the XRF sum spectra were fitted using the PyMCA library (examples shown in Figures S18). The resulting elemental maps were compiled and colored with ImageJ.^68,69^ Further details of detection limit, correction, data fitting, and processing can be found in the Supporting Information, Section S4. Correlation analysis of element fluorescence signal was carried out by cropping and converting regions of interest from the tomographic sections into counts per second per pixel using ImageJ, followed by Spearman’s (*r*_s_) analysis using SciPy.^70^

Additionally, U L_III_-edge (17.163 keV) XANES spectra of the UNP and a uranyl nitrate salt (uranyl nitrate hexahydrate, Sigma-Aldrich, St. Louis, MO, USA), prepared as dry powder thinly spread on Kapton tape, were collected in fluorescence and transmission mode using 1 eV steps from ∼100 eV below to ∼300 eV above the absorption edge. To improve the signal-to-noise ratio, nine spectra were taken at each point and processed for background subtraction and normalization using the ATHENA software.^71^ The resulting μ-XANES spectra were qualitatively compared with those of the uranyl nitrate salt as well as reference UO_2_ and U_3_O_8_ spectra (UO_2_, U_3_O_8_, Institute of Energy Technology, Kjeller, Norway) that were measured at HASYLAB, beamline L (unpublished data).

## Supporting Information Available

Further details on synthesis and characterization of UNPs and UNP stock suspensions (methods and results); *D*. *magna* culturing, exposure parameters, and sample preparation for XRF analysis; acute toxicity tests and associated results; representative XRF sum spectra; elemental mapping of an unexposed, control daphnia; elemental maps in intensity scale, comparisons with maps in logarithmic scale, and correlation analysis of U and Fe/Zn fluorescence signals (PDF)

## References

[ref1] Waersted F. M.; Riss P. J.; Skipperud L. The Effect of Water Exchange on the Leaching of Alum Shale. Appl. Geochem. 2020, 119, 104610 10.1016/j.apgeochem.2020.104610.

[ref2] Salbu B.; Skipperud L.; Lind O. C. Sources Contributing to Radionuclides in the Environment: With Focus on Radioactive Particles. In Radionuclides in the Environment: Influence of Chemical Speciation and Plant Uptake on Radionuclide Migration; Walther C., Gupta D. K., Eds.; Springer International Publishing: Switzerland, 2015; pp 1–36.

[ref3] Strømman G.; Rosseland B. O.; Skipperud L.; Burkitbaev L. M.; Uralbekov B.; Heier L. S.; Salbu B. Uranium Activity Ratio in Water and Fish from Pit Lakes in Kurday, Kazakhstan and Taboshar, Tajikistan. J. Environ. Radioact. 2013, 123, 71–81. 10.1016/j.jenvrad.2012.05.014. 22739115

[ref4] Wang Y.; Bagnoud A.; Suvorova E.; McGivney E.; Chesaux L.; Phrommavanh V.; Descostes M.; Bernier-Latmani R. Geochemical Control on Uranium(IV) Mobility in a Mining-Impacted Wetland. Environ. Sci. Technol. 2014, 48 (17), 10062–10070. 10.1021/es501556d. 25050937

[ref5] Kashparov V. A. Hot particles at Chernobyl. Environ. Sci. Pollut. Res. 2003, 21–30.

[ref6] Lind O. C.; Tschiersch J.; Salbu B. Nanometer-Micrometer Sized Depleted Uranium (DU) Particles in the Environment. J. Environ. Radioact. 2020, 211, 106077 10.1016/j.jenvrad.2019.106077. 31677431

[ref7] Hasan S.; Ghosh T. K. Synthesis of Uranium Oxide Nanoparticles in Aqueous Solutions. Nucl. Technol. 2011, 173 (3), 310–317. 10.13182/NT11-A11664.

[ref8] Salbu B.; Kashparov V.; Lind O. C.; Garcia-Tenorio R.; Johansen M. P.; Child D. P.; Roos P.; Sancho C. Challenges Associated with the Behaviour of Radioactive Particles in the Environment. J. Environ. Radioact. 2018, 186, 101–115. 10.1016/j.jenvrad.2017.09.001. 28941957

[ref9] Kashparov V. A.; Ahamdach N.; Zvarich S. I.; Yoschenko V. I.; Maloshtan I. M.; Dewiere L. Kinetics of Dissolution of Chernobyl Fuel Particles in Soil in Natural Conditions. J. Environ. Radioact. 2004, 72 (3), 335–353. 10.1016/j.jenvrad.2003.08.002. 14972414

[ref10] Suzuki Y.; Kelly S. D.; Kemner K. M.; Banfield J. F. Nanometre-Size Products of Uranium Bioreduction. Nature 2002, 419 (6903), 134–134. 10.1038/419134a. 12226656

[ref11] Bargar J. R.; Bernier-Latmani R.; Giammar D. E.; Tebo B. M. Biogenic Uraninite Nanoparticles and Their Importance for Uranium Remediation. Elements 2008, 4 (6), 407–412. 10.2113/gselements.4.6.407.

[ref12] Guarnieri D.; Sabella S.; Muscetti O.; Belli V.; Malvindi M. A.; Fusco S.; De Luca E.; Pompa P. P.; Netti P. A. Transport Across the Cell-Membrane Dictates Nanoparticle Fate and Toxicity: a New Paradigm in Nanotoxicology. Nanoscale 2014, 6 (17), 10264–10273. 10.1039/C4NR02008A. 25061814

[ref13] Sheppard S. C.; Sheppard M. I.; Gallerand M.-O.; Sanipelli B. Derivation of Ecotoxicity Thresholds for Uranium. J. Environ. Radioact. 2005, 79 (1), 55–83. 10.1016/j.jenvrad.2004.05.015. 15571876

[ref14] Guidelines for Drinking-Water Quality: Fourth ed. Incorporating the First and Second Addenda; World Health Organization: Geneva; 2022; pp 478–480. 35417116

[ref15] Salbu B.; Burkitbaev M.; Strømman G.; Shishkov I.; Kayukov P.; Uralbekov B.; Rosseland B. O. Environmental Impact Assessment of Radionuclides and Trace Elements at the Kurday U Mining Site, Kazakhstan. J. Environ. Radioact. 2013, 123, 14–27. 10.1016/j.jenvrad.2012.05.001. 22789313

[ref16] Ebert D. Ecology, Epidemiology, and Evolution of Parasitism in Daphnia; National Library of Medicine (USA), National Center for Biotechnology Information: Bethesda, MD, USA, 2005.

[ref17] Stollewerk A. The Water Flea Daphnia - a ’New’ Model System for Ecology and Evolution?. J. Biol. 2010, 9 (2), 21 10.1186/jbiol212. 20478012 PMC2871515

[ref18] Poston T. M.; Hanf R. W.; Simmons M. A. Toxicity of Uranium to *Daphnia magna*. Water, Air, and Soil Pollut. 1984, 22 (3), 289–298. 10.1007/BF00159350.

[ref19] Barata C.; Baird D. J.; Markich S. J. Influence of Genetic and Environmental Factors on the Tolerance of *Daphnia magna* Straus to Essential and Non-Essential Metals. Aquat. Toxicol. 1998, 42 (2), 115–137. 10.1016/S0166-445X(98)00039-3.

[ref20] Scheibener S.; Song Y.; Tollefsen K. E.; Salbu B.; Teien H.-C. Uranium Accumulation and Toxicokinetics in the Crustacean *Daphnia magna* Provide Perspective to Toxicodynamic Responses. Aquat. Toxicol. 2021, 235, 105836 10.1016/j.aquatox.2021.105836. 33932687

[ref21] Rossbach L. M.; Brede D. A.; Nuyts G.; Cagno S.; Olsson R. M. S.; Oughton D. H.; Falkenberg G.; Janssens K.; Lind O. C. Synchrotron XRF Analysis Identifies Cerium Accumulation Colocalized with Pharyngeal Deformities in CeO2 NP-Exposed *Caenorhabditis elegans*. Environ. Sci. Technol. 2022, 56 (8), 5081–5089. 10.1021/acs.est.1c08509. 35378039 PMC9022427

[ref22] Wang W.-X. Bioimaging of Metals in Environmental Toxicological Studies: Linking Localization and Functionality. Crit. Rev. Environ. Sci. Technol. 2022, 52, 1–31. 10.1080/10643389.2021.1934368.

[ref23] Pushie M. J.; Pickering I. J.; Korbas M.; Hackett M. J.; George G. N. Elemental and Chemically Specific X-ray Fluorescence Imaging of Biological Systems. Chem. Rev. 2014, 114 (17), 8499–8541. 10.1021/cr4007297. 25102317 PMC4160287

[ref24] Jackson B. P.; Pace H. E.; Lanzirotti A.; Smith R.; Ranville J. F. Synchrotron X-ray 2D and 3D Elemental Imaging of CdSe/ZnS Quantum Dot Nanoparticles in *Daphnia magna*. Anal. Bioanal. Chem. 2009, 394 (3), 911–917. 10.1007/s00216-009-2768-y. 19340415

[ref25] Caumette G.; Koch I.; Moriarty M.; Reimer K. J. Arsenic Distribution and Speciation in *Daphnia pulex*. Sci. Total Environ. 2012, 432, 243–250. 10.1016/j.scitotenv.2012.05.050. 22750169

[ref26] Fouqueray M.; Dufils B.; Vollat B.; Chaurand P.; Botta C.; Abacci K.; Labille J.; Rose J.; Garric J. Effects of Aged TiO2 Nanomaterial from Sunscreen on *Daphnia magna* Exposed by Dietary Route. Environ. Pollut. 2012, 163, 55–61. 10.1016/j.envpol.2011.11.035. 22325431

[ref27] Acharya C.; Blindauer C. A. Unexpected Interactions of the Cyanobacterial Metallothionein SmtA with Uranium. Inorg. Chem. 2016, 55 (4), 1505–1515. 10.1021/acs.inorgchem.5b02327. 26808269

[ref28] Hao Y.; Huang J.; Liu C.; Li H.; Liu J.; Zeng Y.; Yang Z.; Li R. Differential Protein Expression in Metallothionein Protection from Depleted Uranium-Induced Nephrotoxicity. Sci. Rep. 2016, 6 (1), 38942 10.1038/srep38942. 27966587 PMC5155243

[ref29] Massarin S.; Beaudouin R.; Zeman F.; Floriani M.; Gilbin R.; Alonzo F.; Pery A. R. R. Biology-Based Modeling To Analyze Uranium Toxicity Data on *Daphnia magna* in a Multigeneration Study. Environ. Sci. Technol. 2011, 45 (9), 4151–4158. 10.1021/es104082e. 21469640

[ref30] De Samber B.; Silversmit G.; Evens R.; De Schamphelaere K.; Janssen C.; Masschaele B.; Van Hoorebeke L.; Balcaen L.; Vanhaecke F.; Falkenberg G.; et al. Three-Dimensional Elemental Imaging by Means of Synchrotron Radiation Micro-XRF: Developments and Applications in Environmental Chemistry. Anal. Bioanal. Chem. 2008, 390 (1), 267–271. 10.1007/s00216-007-1694-0. 17989960

[ref31] Van Malderen S. J. M.; Laforce B.; Van Acker T.; Nys C.; De Rijcke M.; de Rycke R.; De Bruyne M.; Boone M. N.; De Schamphelaere K.; Borovinskaya O.; et al. Three-Dimensional Reconstruction of the Tissue-Specific Multielemental Distribution within Ceriodaphnia dubia *via* Multimodal Registration Using Laser Ablation ICP-Mass Spectrometry and X-ray Spectroscopic Techniques. Anal. Chem. 2017, 89 (7), 4161–4168. 10.1021/acs.analchem.7b00111. 28256828

[ref32] Teeguarden J. G.; Tan Y.-M.; Edwards S. W.; Leonard J. A.; Anderson K. A.; Corley R. A.; Kile M. L.; Simonich S. M.; Stone D.; Tanguay R. L.; et al. Completing the Link between Exposure Science and Toxicology for Improved Environmental Health Decision Making: The Aggregate Exposure Pathway Framework. Environ. Sci. Technol. 2016, 50 (9), 4579–4586. 10.1021/acs.est.5b05311. 26759916 PMC4854780

[ref33] Tan Y. M.; Leonard J. A.; Edwards S.; Teeguarden J.; Egeghy P. Refining the Aggregate Exposure Pathway. Environ. Sci.-Process Impacts 2018, 20 (3), 428–436. 10.1039/C8EM00018B. 29465734 PMC5909835

[ref34] Handy R. D.; von der Kammer F.; Lead J. R.; Hassellöv M.; Owen R.; Crane M. The Ecotoxicology and Chemistry of Manufactured Nanoparticles. Ecotoxicology 2008, 17 (4), 287–314. 10.1007/s10646-008-0199-8. 18351458

[ref35] Lofts S.; Fevrier L.; Horemans N.; Gilbin R.; Bruggeman C.; Vandenhove H. Assessment of Co-Contaminant Effects on Uranium and Thorium Speciation in Freshwater using Geochemical Modelling. J.l Environ. Radioact. 2015, 149, 99–109. 10.1016/j.jenvrad.2015.07.011. 26225834

[ref36] Markich S. J. Uranium Speciation and Bioavailability in Aquatic Systems: An Overview. Sci. Wor. J. 2002, 2 (2), 707–729. 10.1100/tsw.2002.130. PMC6009715 12805996

[ref37] Goulet R. R.; Fortin C.; Spry D. J. 8 - Uranium. In Fish Physiology; Wood C. M., Farrell A. P., Brauner C. J., Eds.; Academic Press: London, 2011; Vol. 31, pp 391–428.

[ref38] USEPA. Methods for Measuring the Acute Toxicity of Effluents and Receiving Waters to Freshwater and Marine Organisms. In EPA-821-R-02-012; 2002; pp 33–34.

[ref39] Zeman F. A.; Gilbin R.; Alonzo F.; Lecomte-Pradines C.; Garnier-Laplace J.; Aliaume C. Effects of Waterborne Uranium on Survival, Growth, Reproduction and Physiological Processes of the Freshwater Cladoceran *Daphnia magna*. Aquat. Toxicol. 2008, 86 (3), 370–378. 10.1016/j.aquatox.2007.11.018. 18221798

[ref40] Byrnes I.; Rossbach L. M.; Jaroszewicz J.; Grolimund D.; Ferreira Sanchez D.; Gomez-Gonzalez M. A.; Nuyts G.; Reinoso-Maset E.; Janssens K.; Salbu B.; Brede D. A.; Lind O. C. Synchrotron XRF and Histological Analyses Identify Damage to Digestive Tract of Uranium NP-Exposed *Daphnia magna*. Environ. Sci. Technol. 2023, 57 (2), 1071–1079. 10.1021/acs.est.2c07174. 36598768 PMC9850915

[ref41] Fortin C.; Dutels L.; Garnier-Laplace J. Uranium Complexation and Uptake by a Green Alga in Relation to Chemical Speciation: The Importance of the Free Uranyl Ion. Environ. Toxicol. Chem. 2004, 23 (4), 974–981. 10.1897/03-90. 15095894

[ref42] Laforsch C.; Tollrian R. A New Preparation Technique of Daphnids for Scanning Electron Microscopy using Hexamethyldisilazane. Archiv für Hydrobiologie 2000, 149, 587–596. 10.1127/archiv-hydrobiol/149/2000/587.

[ref43] Kikuchi S. The Fine Structure of the Gill Epithelium of a Fresh-Water Flea, *Daphnia magna* (Crustacea: Phyllopoda) and Changes Associated with Acclimation to Various Salinities. Cell Tissue Res. 1983, 229 (2), 253–268. 10.1007/BF00214974. 6850746

[ref44] De Samber B.; De Schamphelaere K. A. C.; Janssen C. R.; Vekemans B.; De Rycke R.; Martinez-Criado G.; Tucoulou R.; Cloetens P.; Vincze L. Hard X-ray Nanoprobe Investigations of the Subtissue Metal Distributions within *Daphnia magna*. Anal. Bioanal. Chem. 2013, 405 (18), 6061–6068. 10.1007/s00216-013-7019-6. 23681201

[ref45] Goldmann T.; Becher B.; Wiedorn K. H.; Pirow R.; Deutschbein M. E.; Vollmer E.; Paul R. J. Epipodite and Fat Cells As Sites of Hemoglobin Synthesis in the Branchiopod Crustacean *Daphnia magna*. Histochem. Cell Biol. 1999, 112 (5), 335–339. 10.1007/PL00007905. 10603072

[ref46] Gophen M.; Geller W. Filter Mesh Size and Food Particle Uptake by Daphnia. Oecologia 1984, 64 (3), 408–412. 10.1007/BF00379140. 28311458

[ref47] Smirnov N. N. 7 - Excretion. In Physiology of the Cladocera; Academic Press (USA), Cambridge (MA), 2017; pp 113–114.

[ref48] Duneau D.; Ebert D. The Role of Moulting in Parasite Defence. Proc. R. Soc. B 2012, 279 (1740), 3049–3054. 10.1098/rspb.2012.0407. PMC3385484 22496187

[ref49] van der Zande M.; Kokalj A. J.; Spurgeon D. J.; Loureiro S.; Silva P. V.; Khodaparast Z.; Drobne D.; Clark N. J.; van den Brink N. W.; Baccaro M.; et al. The Gut Barrier and the Fate of Engineered Nanomaterials: a View from Comparative Physiology. Environ.-Sci. Nano 2020, 7 (7), 1874–1898. 10.1039/D0EN00174K.

[ref50] Vicente-Vicente L.; Quiros Y.; Pérez-Barriocanal F.; López-Novoa J. M.; López-Hernández F. J.; Morales A. I. Nephrotoxicity of Uranium: Pathophysiological, Diagnostic and Therapeutic Perspectives. Toxicol. Sci. 2010, 118 (2), 324–347. 10.1093/toxsci/kfq178. 20554698

[ref51] Plaire D.; Bourdineaud J.-P.; Alonzo A.; Camilleri V.; Garcia-Sanchez L.; Adam-Guillermin C.; Alonzo F. Transmission of DNA Damage and Reprotoxic Effects Over Two Generations of Daphnia magna Exposed to Uranium. Comp. Biochem. Physiol., Part C: Toxicol. Pharmacol. 2013, 158 (4), 231–243. 10.1016/j.cbpc.2013.09.001. 24035969

[ref52] Massarin S.; Alonzo F.; Garcia-Sanchez L.; Gilbin R.; Garnier-Laplace J.; Poggiale J.-C. Effects of Chronic Uranium Exposure on Life History and Physiology of *Daphnia magna* Over Three Successive Generations. Aquat. Toxicol. 2010, 99 (3), 309–319. 10.1016/j.aquatox.2010.05.006. 20646767

[ref53] Lee D.; Nah J. S.; Yoon J.; Kim W.; Rhee K. Live Observation of the Oviposition Process in *Daphnia magna*. PLoS One 2019, 14 (11), e0224388 10.1371/journal.pone.0224388. 31682612 PMC6827901

[ref54] Yan N.; Tsim S. M. J.; He X.; Tang B. Z.; Wang W.-X. Direct Visualization and Quantification of Maternal Transfer of Silver Nanoparticles in Zooplankton. Environ. Sci. Technol. 2020, 54 (17), 10763–10771. 10.1021/acs.est.0c03228. 32786596

[ref55] Aladin N.; Potts W. Osmoregulatory Capacity of the Cladocera. J. Comp. Physiol., B 1995, 164 (8), 671–683. 10.1007/BF00389810.

[ref56] Charmantier G.; Charmantier-Daures M. Ontogeny of Osmoregulation in Crustaceans: The Embryonic Phase1. Am. Zool. 2015, 41 (5), 1078–1089. 10.1093/icb/41.5.1078.

[ref57] Morris C.; O’Donnell M. Multiple Functions of Ion Transport by the Nuchal Organ in Embryos and Neonates of the Freshwater Branchiopod Crustacean *Daphnia magna*. J. Exp. Biol. 2019, 222 (22), jeb211128 10.1242/jeb.211128. 31645374

[ref58] Mittmann B.; Ungerer P.; Klann M.; Stollewerk A.; Wolff C. Development and Staging of the Water Flea *Daphnia magna* (Straus, 1820; Cladocera, Daphniidae) based on morphological landmarks. EvoDevo 2014, 5 (1), 12 10.1186/2041-9139-5-12. 24641948 PMC4108089

[ref59] Brun N. R.; Beenakker M. M. T.; Hunting E. R.; Ebert D.; Vijver M. G. Brood Pouch-Mediated Polystyrene Nanoparticle Uptake During *Daphnia magna* Embryogenesis. Nanotoxicology 2017, 11 (8), 1059–1069. 10.1080/17435390.2017.1391344. 29083253

[ref60] Pavelkova T.; Cuba V.; Sebesta F. Photo-Induced Low Temperature Synthesis of Nanocrystalline UO_2_, ThO_2_ and mixed UO_2_-ThO_2_ Oxides. J. Nucl. Mater. 2013, 442 (1–3), 29–32. 10.1016/j.jnucmat.2013.08.016.

[ref61] Pavelková T.; Čuba V.; De Visser-Týnová E.; Ekberg C.; Persson I. Preparation of UO_2_, ThO_2_ and (Th,U)O_2_ Pellets from Photochemically-Prepared Nano-Powders. J. Nucl. Mater. 2016, 469, 57–61. 10.1016/j.jnucmat.2015.11.041.

[ref62] OECD. Test No. 202: *Daphnia* sp. Acute Immobilisation Test, OECD Guidelines for the Testing of Chemicals, Section 2, OECD Publishing: Paris, 2004.

[ref63] Charles S.; Veber P.; Delignette-Muller M. L. MOSAIC: a Web-Interface for Statistical Analyses in Ecotoxicology. Environ. Sci. Pollut. Res. 2018, 25 (12), 11295–11302. 10.1007/s11356-017-9809-4. 28842838

[ref64] Morse: Modelling Tools for Reproduction and Survival Data in Ecotoxicology; 2016, https://CRAN.R-project.org/package=morse (accessed Jan 13, 2021).

[ref65] Tan L.-Y.; Huang B.; Xu S.; Wei Z.-B.; Yang L.-Y.; Miao A.-J. TiO_2_ Nanoparticle Uptake by the Water Flea *Daphnia magna via* Different Routes is Calcium-Dependent. Environ. Sci. Technol. 2016, 50 (14), 7799–7807. 10.1021/acs.est.6b01645. 27359244

[ref66] van Aarle W.; Palenstijn W. J.; De Beenhouwer J.; Altantzis T.; Bals S.; Batenburg K. J.; Sijbers J. The ASTRA Toolbox: A Platform for Advanced Algorithm Development in Electron Tomography. Ultramicroscopy 2015, 157, 35–47. 10.1016/j.ultramic.2015.05.002. 26057688

[ref67] van Aarle W.; Palenstijn W. J.; Cant J.; Janssens E.; Bleichrodt F.; Dabravolski A.; De Beenhouwer J.; Batenburg K. J.; Sijbers J. Fast and Flexible X-ray Tomography Using the ASTRA Toolbox. Opt. Express 2016, 24 (22), 25129–25147. 10.1364/OE.24.025129. 27828452

[ref68] Schindelin J.; Rueden C. T.; Hiner M. C.; Eliceiri K. W. The ImageJ ecosystem: An Open Platform for Biomedical Image Analysis. Mol. Reprod. Dev. 2015, 82 (7–8), 518–529. 10.1002/mrd.22489. 26153368 PMC5428984

[ref69] Solé V. A.; Papillon E.; Cotte M.; Walter P.; Susini J. A Multiplatform Code for the Analysis of Energy-Dispersive X-ray Fluorescence Spectra. Spectrochim. Acta, Part B 2007, 62 (1), 63–68. 10.1016/j.sab.2006.12.002.

[ref70] Virtanen P.; Gommers R.; Oliphant T. E.; Haberland M.; Reddy T.; Cournapeau D.; Burovski E.; Peterson P.; Weckesser W.; Bright J.; et al. SciPy 1.0: fundamental algorithms for scientific computing in Python. Nat. Methods 2020, 17 (3), 261–272. 10.1038/s41592-019-0686-2. 32015543 PMC7056644

[ref71] Ravel B.; Newville M. ATHENA, ARTEMIS, HEPHAESTUS: Data Analysis for X-ray Absorption Spectroscopy Using IFEFFIT. J. Synchrotron Radiat. 2005, 12 (4), 537–541. 10.1107/S0909049505012719. 15968136

