# Supplementary material for: Synchrotron-Based X-ray Fluorescence Imaging Elucidates Uranium Toxicokinetics in Daphnia magna
Source: ACS Nano. 2023 Mar 15;17(6):5296–305. doi: 10.1021/acsnano.2c06111 (PMC10062025; doi:10.1021/acsnano.2c06111)
Supplement: Supplementary file 1 — nn2c06111_si_001.pdf [file nn2c06111_si_001.pdf]

Supporting Information for

Synchrotron Based X-ray Fluorescence Imaging Elucidates Uranium Toxicokinetics in *Daphnia magna*

Ian Byrnes[1*], Lisa Magdalena Rossbach[1], Dag Anders Brede[1], Daniel Grolimund[2], Dario Ferreira Sanchez[2], Gert Nuyts[3], Vaclav Cuba[4], Estela Reinoso-Maset[1], Brit Salbu[1], Koen Janssens[3], Deborah Oughton[1], Shane Scheibener[1], Hans-Christian Teien[1], Ole Christian Lind[1*]

[1] Norwegian University of Life Sciences, Centre for Environmental Radioactivity (CERAD), Faculty of Environmental Sciences and Natural Resource Management, P.O. Box 5003, 1433 Ås, Norway

[2] Swiss Light Source, Paul Scherrer Institute (PSI), 5232 Villigen, Switzerland

[3] University of Antwerp, AXIS Group, NANOlab Center of Excellence, Department of Physics, Groenenborgerlaan 171, 2020 Antwerp, Belgium

[4] Czech Technical University in Prague, Faculty of Nuclear Sciences and Physical Engineering, Brehova 7, Prague 1, Czech Republic

*Corresponding Authors

Phone: +47 67 23 18 95

Email Address: ian.byrnes@nmbu.no, ole-christian.lind@nmbu.no

Address: Norwegian University of Life Sciences, Centre for Environmental Radioactivity (CERAD), Faculty of Environmental Sciences and Natural Resource Management, P.O. Box 5003, 1433 Ås, Norway

ASC Nano

March 2023

Summary

The following supplementary materials include description of experimental methods and additional results.

Experimental Methods

S1. Uranium Nanoparticle Synthesis and Characterization

S2. Uranium Nanoparticle Stock Suspension Characterization

S3. *Daphnia magna* Culturing and Acute Toxicity Tests

S4. Synchrotron X-ray Flourescence Analysis and Image Processing

Results

Figure S1: XRD Analysis of Dry UNP Powders

Figure S2: µ-XANES of Dry UNP Powders

Figure S3: TEM Analysis of UNP Stock Solution

Table S1: Exposure Experiment Parameters

Table S2: Elemental Composition of the UNP Stock Solution

Figure S4: Uranium Size Distributions of Exposure Media

Figure S5: TEM Analysis of U_{Ref} Solutions

Figure S6: *Daphnia magna* Survival Curves

Table S3: 48 h LC_{50} and LC_{10} Values

Figure S7: Total Uranium Body Burden in *Daphnia magna*

Figure S8: Daphnid Survival as a Function of Uranium Body Burden

Figure S9: Chemically Dried *Daphnia magna* Mounting

Figure S10: Uranium Map Comparison Using Linear and Logarithmic Scale

Figure S11: Uranium Removal by Sample Preparation

Figure S12: Epipodite Uranium Distributions with Intensity Scale

Figure S13: Correlation Analysis of Daphnid Epipodite (U_{Ref})

Figure S14: Correlation Analysis of Daphnid Epipodite (UNP)

Figure S15: Uranium Tomographic Sections with Intensity Scale

Figure S16: Elemental Mapping of Control Sample

Figure S17: Tomographic Section of UNP Exposed *Daphnia magna*

Table S4: Reproductive Observation

Figure S18: XRF Sum Spectra

Experimental Methods

S1. Uranium Nanoparticle Synthesis and Characterization

All chemicals, unless stated otherwise, were of analytical grade (Merck, Czech Republic). Deionized water was used for the preparation of aqueous solutions. Uranyl nitrate (Lachema, CSSR) was annealed at 1200 °C for 2 h. The purity of U_3O_8 was evaluated by X-ray powder diffraction (XRD).

A reaction mixture was prepared by dissolution of U_3O_8 (5.614 g, 6.7 mmol) in 4 mL of concentrated nitric acid (65 %), which corresponds to 10 % excess of nitric acid. The dissolution was accompanied by the release of nitric fumes. Following the complete dissolution of U_3O_8, the resulting solution was diluted with deionized water to prevent precipitation after addition of propan-2-ol. Finally, 200 mL of propan-2-ol (10 $\%_{vol}$) was added and the solution was again diluted with deionized water to a total volume of 2 L. The resulting concentration of UO_2^{2+} was 10 mM. This solution was stirred with a magnetic stirrer and irradiated for 150 min in a photochemical reactor with immersed quartz-protected low-pressure mercury lamps (variable power input: 400 W (nominal value), wavelength 254 nm; Philips TUV 25WP SE) and cooled by air-ventilators. The formed dark grey product was separated from solution by centrifugation, washed in ethanol in order to remove synthesis residues, and subsequently air dried at 40 °C.

The final material was also characterized by XRD using a Rigaku MiniFlex 600 (Ni-filtered Cu-Kα1,2 radiation) equipped with a NaI:Tl scintillation detector. XRD patterns were compared to the relevant records in the ICDD PDF-2 database (version 2013). The angular range was 10° – 80°, with a step of 0.02° and a scanning speed of 2°/min.

S2. Uranium Nanoparticle Suspension and Stock Characterization

Stocks were prepared (1.0 g L^{-1}) by weighing UNPs into 20 mL glass vials, applying a dispersion agent (1 % v/v polyoxyethelene glycerol triolate), and then dispersing them in 10 mL N_2-purged ddH_2O (15 MΩ cm). A 400-W Branson Sonifier S-450D (Branson Ultrasonics) equipped with a standard 13 mm disruptor tip (model 101-147-037) was used to sonify the UNP stocks for 13 min at a 15 % amplitude.

Dynamic Light Scattering Measurements

Zeta-average hydrodynamic diameters and zeta-potentials of the UNP stock suspensions were determined by dynamic light scattering (DLS) using a Malvern Zetasizer ZS (Malvern Instruments Ltd., Worcestershire, United Kingdom) equipped with a 633 nm laser. Zeta-average hydrodynamic diameter measurements were conducted in triplicate, 5 runs each, with autocorrection function of 10 s. Electrophoretic mobility (zeta potential) was determined by Smoluchowski approximations.

Electron Microscopy

Particle crystalline structure and individual sizes were confirmed by high resolution (HR) transmission electron microscopy (TEM), while scanning transmission electron microscopy (STEM) with energy dispersive X-ray spectroscopy (EDS) was used for elemental composition. Immediately following sonication, 10 µL of UNP stock suspension was added to a 400 mesh formvar-carbon film (Agar Scientific Ltd., Essex, United Kingdom) and allowed to air dry. Samples were measured at 200 kV accelerating voltage on a JEOL JEM-2100F equipped with a Gatan Porius 200D CCD camera (JOEL Ltd., Tokyo, Japan). Fluorescent X-rays were collected by an Oxford X-Max-80 SDD EDS detector at a 0.23 srad collection angle.

Uranium Reference Solution Preparation

To compare with the UNP exposure, a U reference (U_{Ref}) solution was prepared from a 1.0 g L^{-1} uranium oxide (U_3O_8) assay and isotopic standard (CRM 129-A, U.S. Department of Energy, Argonne, Illinois, USA). A stock solution of 100 mg U L^{-1} was prepared in ddH_2O and aliqouts of this stock were added directly to empty 50 mL plastic exposure cups (Graduated Polypropylene, VWR, Radnor, PA) to result in an exposure solution with a given U concentration. The solutions were evaporated to dryness and, 24 h prior to the start of the daphnia exposure, re-dissolved with 25 mL of exposure media (i.e., MHRW at pH 6.8).

Particulate and colloidal U species in the U_{Ref} solutions were examined by TEM analysis. A 5 mg U L^{-1} U_{Ref} solution was prepared in MHRW (pH 6.8) by the same procedure described previously. After 24 h, the solution was sampled by filtering the 50 mL solution

through a 3 kDa filter (Amicon Millipore, Billerica, MA) in 15 mL increments at 5,000 *g* for 90 min each. Next the filter cartidge was washed using 10 mL of ddH_2O (15 MΩ cm) and centrifuged again at 5,000 *g* for 90 min. Samples for TEM analysis were taken from 10 µL of retentate found in the bottom of the filter cartidge and placed onto a 400 mesh lacy carbon film (Agar Scientific Ltd., Essex, United Kingdom) and allowed to air dry. Samples were measured at 200 kV accelerating voltage on a JEOL JEM-2100F equipped with a TVIPS TemCam-XS416 (ES). Fluorescent X-rays were collected by an Oxford X-Max-80 SDD EDS detector at a 0.23 srad collection angle.

Exposure Media Size Fractionation

Size fractionation was used to determine the size distribution of U species at 0, 24, and 48 h after the start of the UNP and U_{Ref} exposures. The size fractions were *particulate* (> 0.45 µm), *colloidal* (3 kDa < x < 0.45 µm), and *low molecular mass species* (LMM, < 3 kDa). In sampled exposures, 1 mL of media was passed through a pre-conditioned (1 mL) 0.45 µm syringe filter (VWR, Radnor, Pennsylvania, United States) and 100 µL was sampled from the filtrate. Next, 400 µL was sampled from the < 0.45 µm solution into a pre-conditioned (300 µL) 3 kDa Amicon cellulose membrane filter (Amicon Millipore, Billerica, MA) and centrifuged at 14,000 *g* for 30 min. From the filtrate, 100 µL was sampled prior to the QQQ-ICP-MS measurement.

Inductively Coupled Plasma Mass Spectrometry

Elemental analysis of exposure solutions, size fractionation samples, and digested daphnid was conducted by triple quadrupole inductively coupled plasma mass spectrometry (ICP-QQQ, Agilent 8900). Aliquots were diluted in 5 % HNO_3 (V/V) 48 h prior to measurement of U. Measurements of digested daphnia had a limit of detection (LOD) of 0.008 µg ^{238}U L^{-1} and a limit of quantification (LOQ) of 0.026 µg ^{238}U L^{-1}. Measurements of digested water samples for media characterization had a LOD of 0.003 µg ^{238}U L^{-1} and a LOQ of 0.009 µg ^{238}U L^{-1} while for daphnid measurements the LOD was 0.008 µg ^{238}U L^{-1} and the LOQ was 0.026 µg ^{238}U L^{-1}.

S3. *Daphnia magna* Culturing and Toxicity Tests

Laboratory cultured *D. magna*, DHI strain (DHI Water & Environment, Hørsholm, Denmark), were reared in M7 media according to OECD guidelines. Cultures were maintained at 20 °C (± 1 °C) with a day-night cycle of 16 h light:8 h darkness while the media was renewed 3 times weekly with neonates removed at those times. Daphnids were fed a diet of concentrated green algae (*Raphidocelis subcapitata*) at a rate of 2.1 × 10^7 cells day^{-1} $daphnid^{-1}$. Synchronized neonates (< 18 h) derived from the second clutch or later were used for exposure experiments.

Acute toxicity tests using < 18 h *D. magna* neonates and < 7 d adults were conducted in US EPA moderately hard reconstituted water (MHRW, pH 6.8, 350 μS cm^{-1}, 20 °C) and 48 h LC_{50} values for UNPs and the U_{Ref} were determined according to OECD *Test No. 202*. The measured exposure concentrations are found in Table S1. Uranium concentrations were determined by ICP-QQQ after 48 h of exposure. After this exposure time, live daphnids were washed three times (MHRW, MHRW, deionized water) and moved to sample preparation. Three individuals were collected for U body burden measurements and digested in 500 μL ultrapure HNO_3 for at least 48 h. In the adult exposure, F0 individuals (n = 3) per sublethal UNP and the U_{ref} exposure groups were placed into clean MHRW with algae feed to observe success of spawning.

S4. Synchrotron X-ray Fluorescence Analysis and Image Processing

The hard X-ray microprobe facility (microXAS – X05LA) at the Swiss Light Source (Paul Scherrer Institute, SLS, Switzerland) was used to evaluate the whole body elemental distribution in prepared *D. magna* samples (Fig. S9). The incident beam size was 1 $μm^2$ using X-rays focused by a Kirkpatrick-Baez (KB) mirror system with an energy of 17.2 keV selected with a fixed-exit double-crystal monochromator. The resulting photon flux was 2 × 10^{10} ph s^{-1}. Daphnid samples were raster scanned with a step size of 5 μm for the whole organism maps and 2 μm for regions of interest (ROI). X-ray fluorescence virtual slices were obtained by computed tomographic analysis *via* line profile projections collected at different orientations over 180°. Fluorescent X-rays were collected using four silicon drift detectors (SDD; Ketek GmbH, Germany) with an 8 μm Be window positioned approximately 2 cm from the sample with equidistant 90° of separation. The dead time

observed in this experiment was below 1 % for all measurements. A dwell time of 200 ms was used for all measurements and the dead time remained below 1 %.

Determination of Detection Limits

A reference standard containing 1.5 µg Cu cm^{-2} (1.4 × 10^{7} atoms µm^{-2}) was measured during the synchrotron experiments and yielded 1370 photon counts s^{-1} for Cu K_{α} and K_{β} lines, integrated over 160 s. As a result, 100 cps was resolvable leading to a detection limit of 1 × 10^{6} Cu atoms µm^{-2}, integrated over 1 s.

Attenuation Correction

The effects of self-absorption were not observed and no attenuation correction was applied in this experiment. The attenuation in denser regions of the daphnid samples, such as some hotspots, never exceeded 5 % absorption of the 17.2 keV beam. Furthermore, the 4 SSDs surrounding the sample, which mostly contained empty space, were combined to minimize the effects of self-absorption.

Spectra Fitting and Image Processing

The resulting sum spectra encompassing the signals from all 4 SSDs for each XRF measurement was opened and fitted using PyMCA. Example sum spectra can be seen in Figure S13 for the UNP (A) and U_{Ref} (B) exposed daphnids, respectively. Upon applying a fit, the resulting TIFF images were exported to ImageJ, where the maps were converted to a logarithmic scale and a Look Up Table (LUT) was applied to color the image. A comparison of the U map in linear and logarithmic scale is shown in Figure S14.

Results

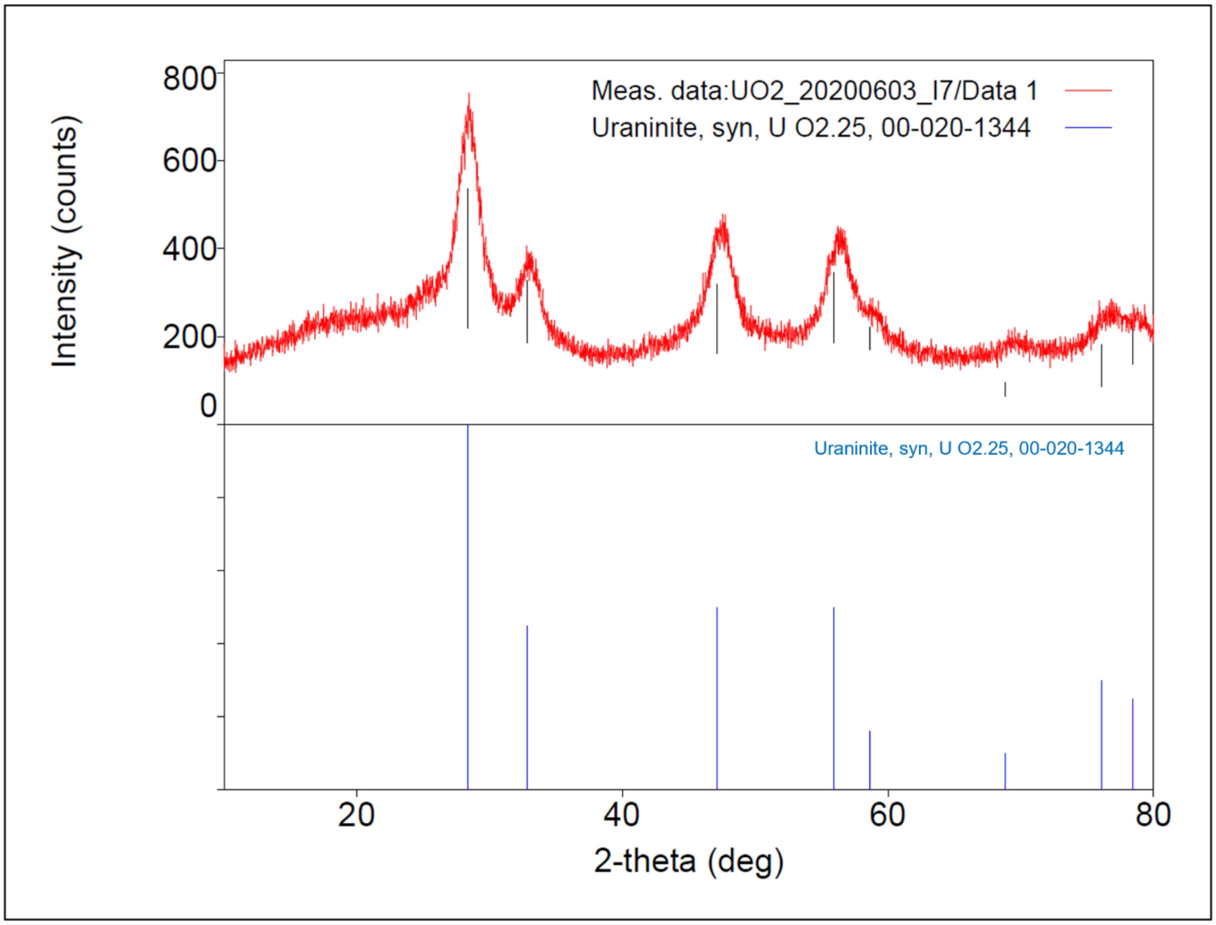

Figure S1: X-ray diffraction patterns of the synthesized UNP powders and a U(IV) reference compound (synthetic uraninite) obtained from the ICDD database.

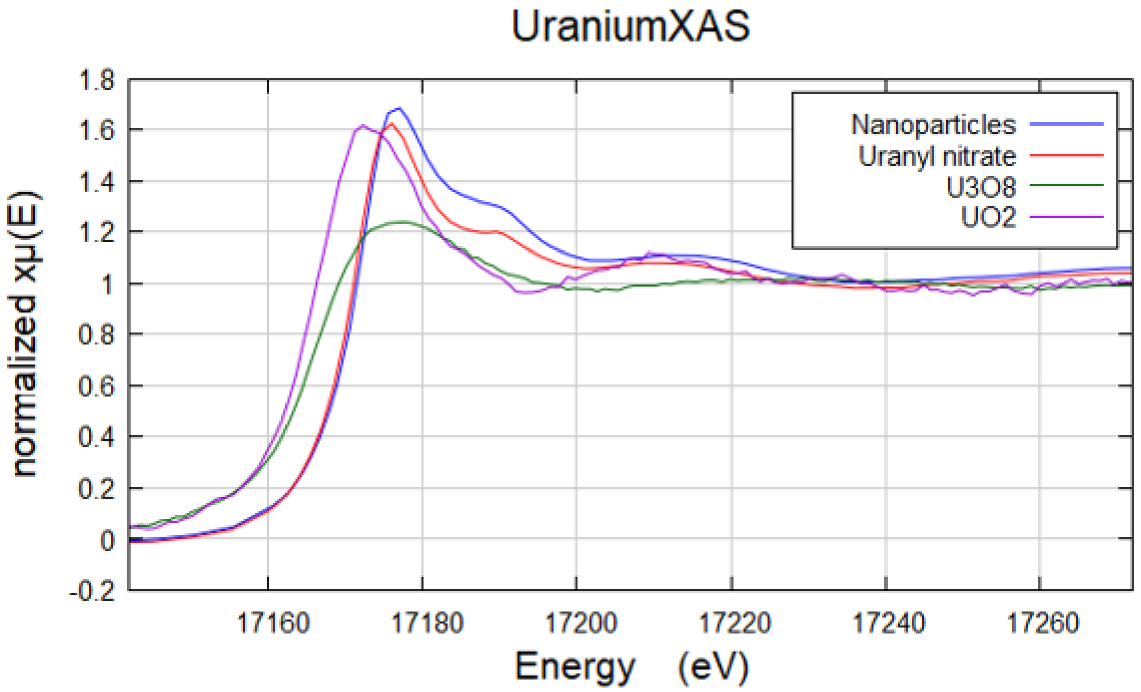

Figure S2: Uranium L_{III}-edge micro-X-ray absorption near edge structure (μ-XANES) spectra of UNP dry powders and uranyl nitrate salts. XANES spectra of UO_2 and U_3O_8 were used as reference compound spectra for comparison, respectively.

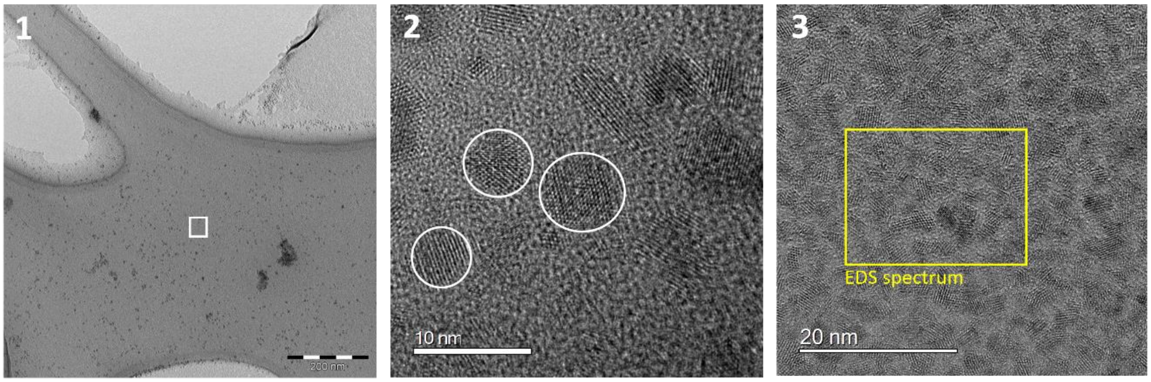

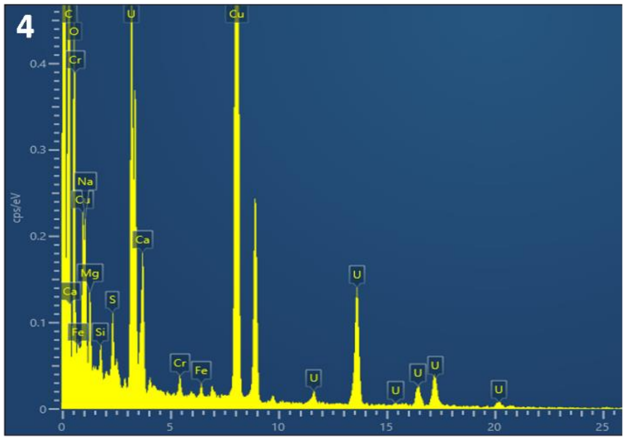

5

Element	Weight %	Atomic %
U	52.67	8.63
O	26.70	65.06
Ca	6.23	6.06
S	2.85	3.46
Mg	3.09	4.96
Na	5.04	8.55
Cr	1.31	0.98
Fe	0.89	0.62
Si	1.21	1.68

Figure S3: Transmission electron microscopy analysis of UNPs in ddH_2O (1 and 2) and in MHRW (3). Bright field TEM image (1) of the UNPs reveals individual particles with some larger aggregates. HR-TEM image (2) of the area within the white square in (1) shows clear lattice fringes of individual particles (white circles, ~5 nm diameter). The area within the yellow square in (3) was analysed by EDS, with the resulting spectrum (4) and associated elemental quantification (5) showing U and O as the major components of the UNPs.

Table S1: Uranium nanoparticle (UNP) suspension details and *D. magna* exposure parameters.

Experiment	Z-average diameter 0 h (nm)	Zeta potential at 0 h (mV)	UNP Measured Exposure Concentrations 48 h (µg U L^{-1})	U_{Ref} Measured Exposure Concentrations 48 h (µg U L^{-1})
Neonates (< 18 h)	205.7 ± 8.1	-9.01	6.51 ± 1.12	8.98 ± 0.79
			28.1 ± 1.8	34.2 ± 2.5
			64.8 ± 4.3	93.3 ± 20.7
			80.3 ± 5.8	111 ± 4
			148 ± 6	188 ± 17
			427 ± 22	258 ± 10
				522 ± 41
				691 ± 9
				1250 ± 141
Adults (< 7 d)	273.3 ± 1.2	-11.8	8.8 ± 1.9	10.8 ± 1.6
			82.5 ± 12.7	91.4 ± 12.9
			112 ± 4	139 ± 15
			161 ± 12	203 ± 7
			195 ± 13	260 ± 13
			388 ± 10	510 ± 48
			591 ± 37	689 ± 29
			781 ± 85	790 ± 42

Table S2: Concentration of the different elements identified in the UNP exposure suspensions used in the neonate and adult daphnids experiments.

	**Li (µg L^{-1})**	**B (µg L^{-1})**	**Ti (µg L^{-1})**	**V (µg L^{-1})**	**Mo (µg L^{-1})**	**Ag (µg L^{-1})**	**Sn (µg L^{-1})**	**Lu (µg L^{-1})**	**U (mg L^{-1})**
**Neonate Experiment**	47.9 ± 2.1	3213 ± 107	1480 ± 618	41.4 ± 7.1	0.79 ± 0.07	19.5 ± 0.4	3.60 ± 0.03	2.19 ± 0.08	665 ± 16
**Adult Experiment**	50.3 ± 0.6	2257 ± 9	373.7 ± 25.4	21.1 ± 1.8	1.32 ± 0.08	17.2 ± 0.9	3.14 ± 0.80	1.80 ± 0.22	708 ± 19

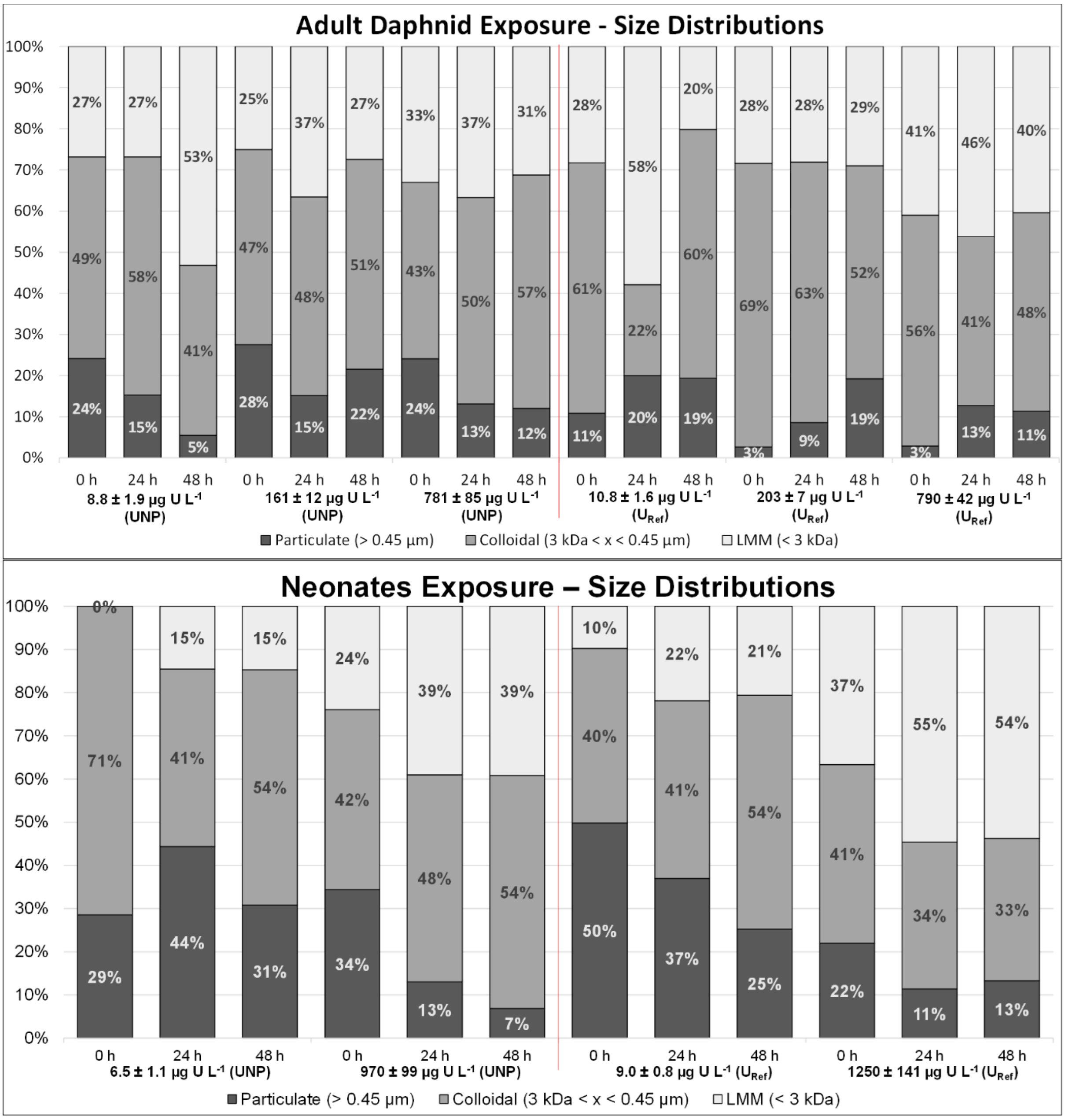

Figure S4: Size distribution of U species in the UNP (*left of red line*) and U_{Ref} (*right of red line*) exposure media solutions of adult daphnids (*top*) and neonates (*bottom*).

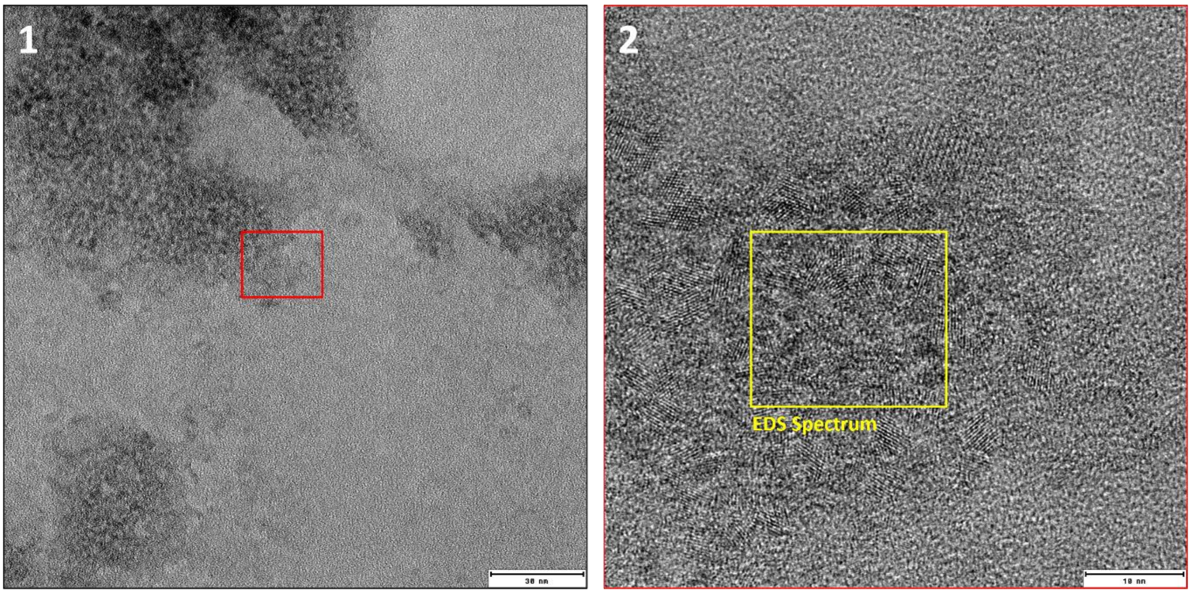

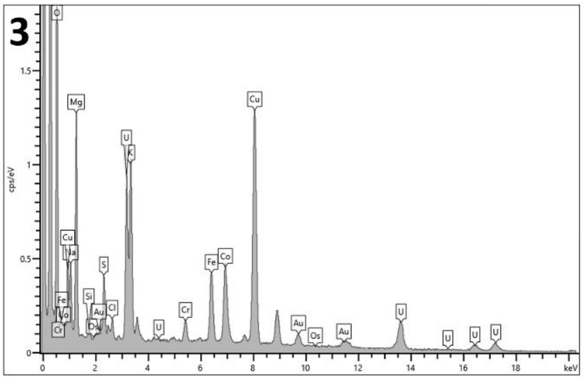

4

Element	Atomic %	Element	Atomic %
O	50.38	Cr	1.54
Na	3.56	Fe	5.43
Mg	16.36	Co	6.05
S	4.18	Au	1.25
Cl	1.27	U	4.06
K	5.93		

Figure S5: Transmission electron microscopy analysis of colloids in the U_{Ref} exposure (MHRW). Bright field TEM image (1) of the U_{Ref} colloids revealed individual particles with crystalline structure. The HR-TEM image (2) of the area within the red square in (1) shows the clear lattice fringes of individual particles (5 – 10 nm diameter). EDS analysis of the area within the yellow square in (2), the resulting spectrum (3) and the associated elemental quantification (4) confirmed the presence of U in the particles.

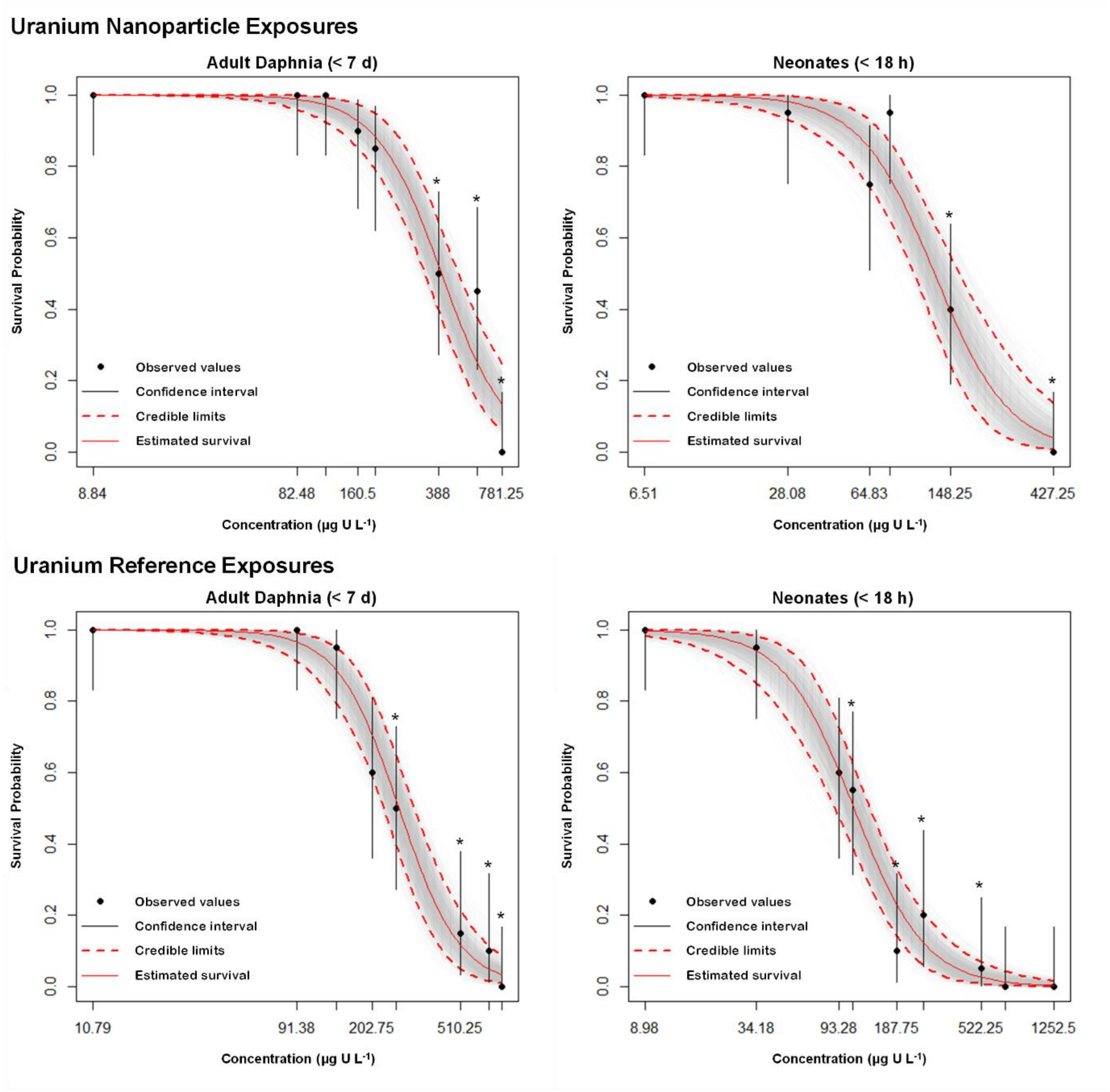

Figure S6: Survival curves for the UNP (*top*) and U_{Ref} (*bottom*) exposures with the modeled survival probability in terms of average measured exposure concentration (µg U L^{-1}). Survival in the adult (< 7 d) (*left*) and neonate (*right*) exposures are presented for each treatment. Input (observed) values are marked by black points with 95 % confidence bars. Fitted survival probability is indicated by the red line, while the grey bands indicate the 95 % credible limits of the model. The confidence interval of all observations was within the model 95 % credible limit. For each exposure, the 48 h LC_{50} and LC_{10} are provided including the 95 % credible limits. Asterisks (*) indicate statistically significant differences compared to the control exposure (ANOVA and non-parametric Kruskal-Wallis (U_{Ref} neonates) tests, $p < 0.05$).

Table S3: Determination of 48 h LC_{50} and LC_{10} in UNP and U_{Ref} exposures of adult daphnids and neonates.

	Adult Daphnia (< 7 d)	Neonates (< 18 h)
**Uranium Nanoparticles**		
LC_{50}	402 µg U L^{-1} [336 - 484]	127 µg U L^{-1} [102 - 163]
LC_{10}	183 µg U L^{-1} [130 - 238]	54.7 µg U L^{-1} [35.7 - 73.4]
**Uranium Reference**		
LC_{50}	268 µg U L^{-1} [229 - 315]	112 µg U L^{-1} [89.5 - 136]
LC_{10}	133 µg U L^{-1} [97.8 - 168]	44.0 µg U L^{-1} [26.5 - 62.0]

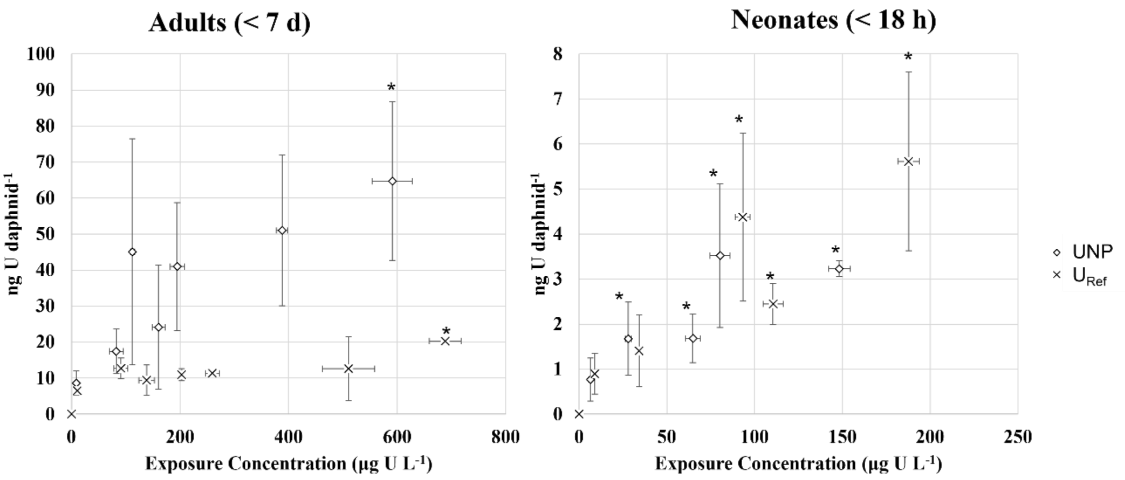

Figure S7: Total U body burden (n = 3) after 48 h acute exposures of neonates (*left*) and adult daphnids (*right*). Asterisks (*) indicate statistically significant differences compared to control exposures (ANOVA test, $p < 0.05$).

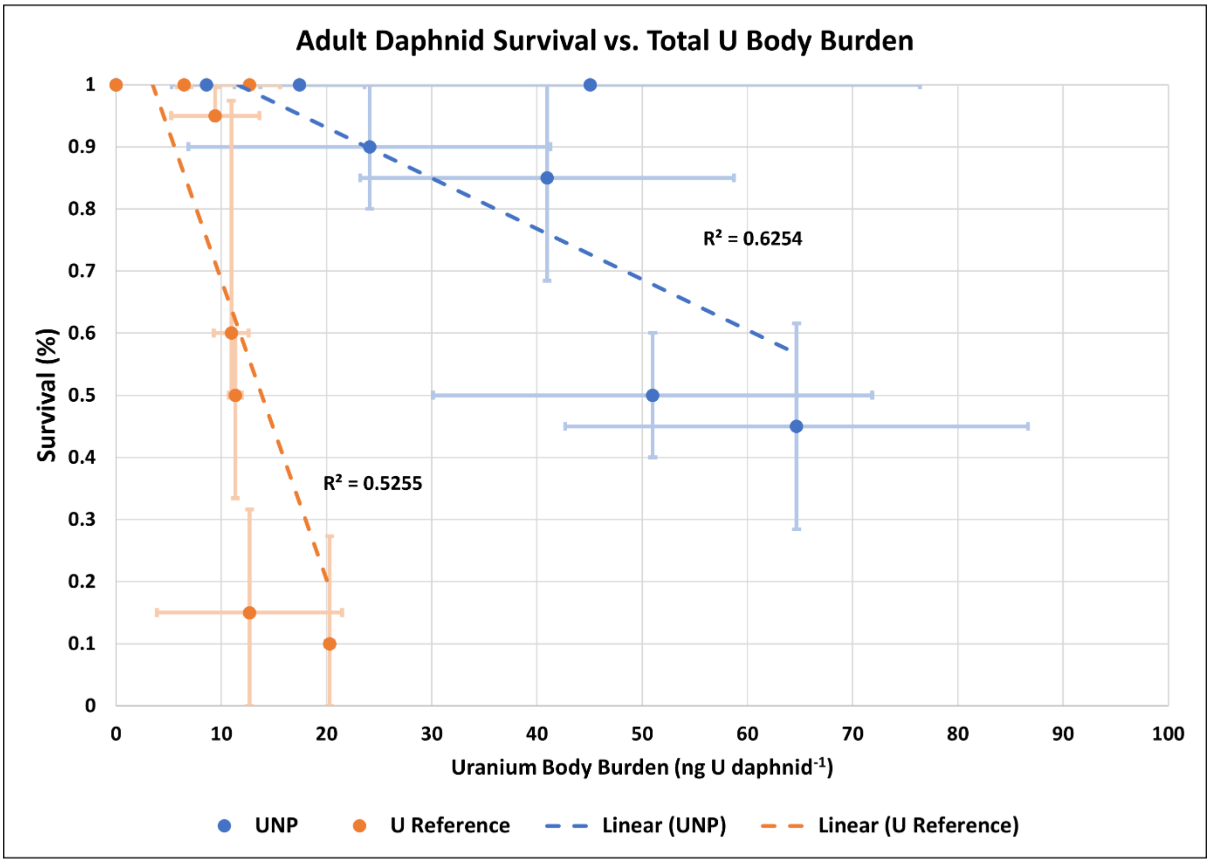

Figure S8: Adult *D. magna* survival as a function of U body burden (ng U daphnid^{-1}). Regression analysis found $p < 0.05$ for both the UNP and the U_{Ref} exposures.

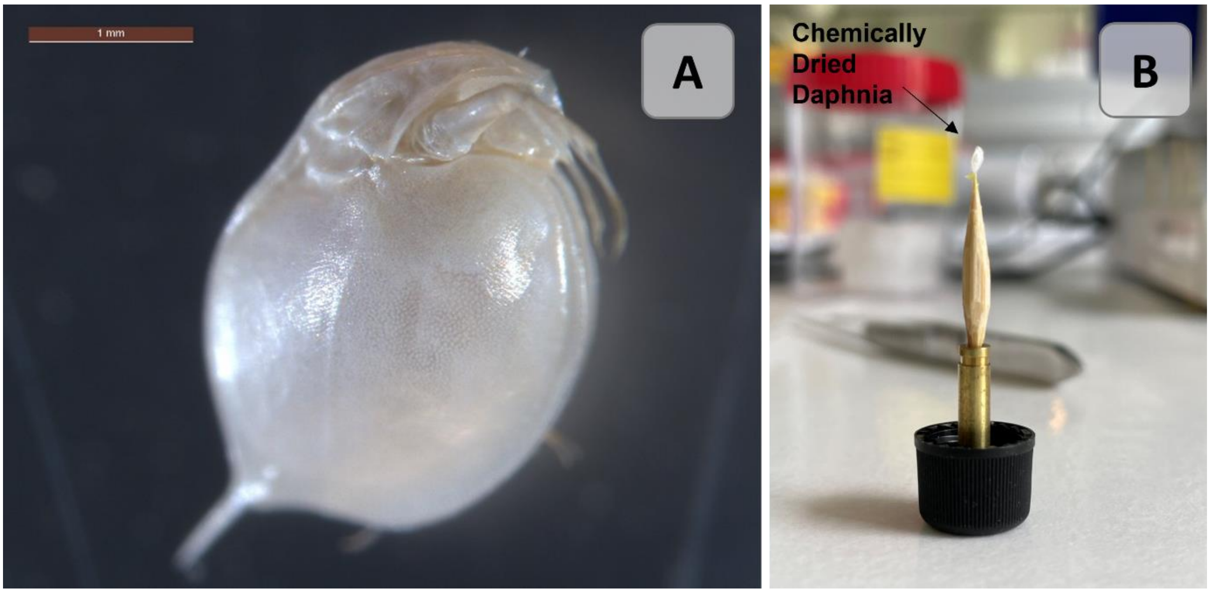

Figure S9: (**A**) Chemically dried *D. magna* and (**B**) sample mounted on the end of a wooden toothpick.

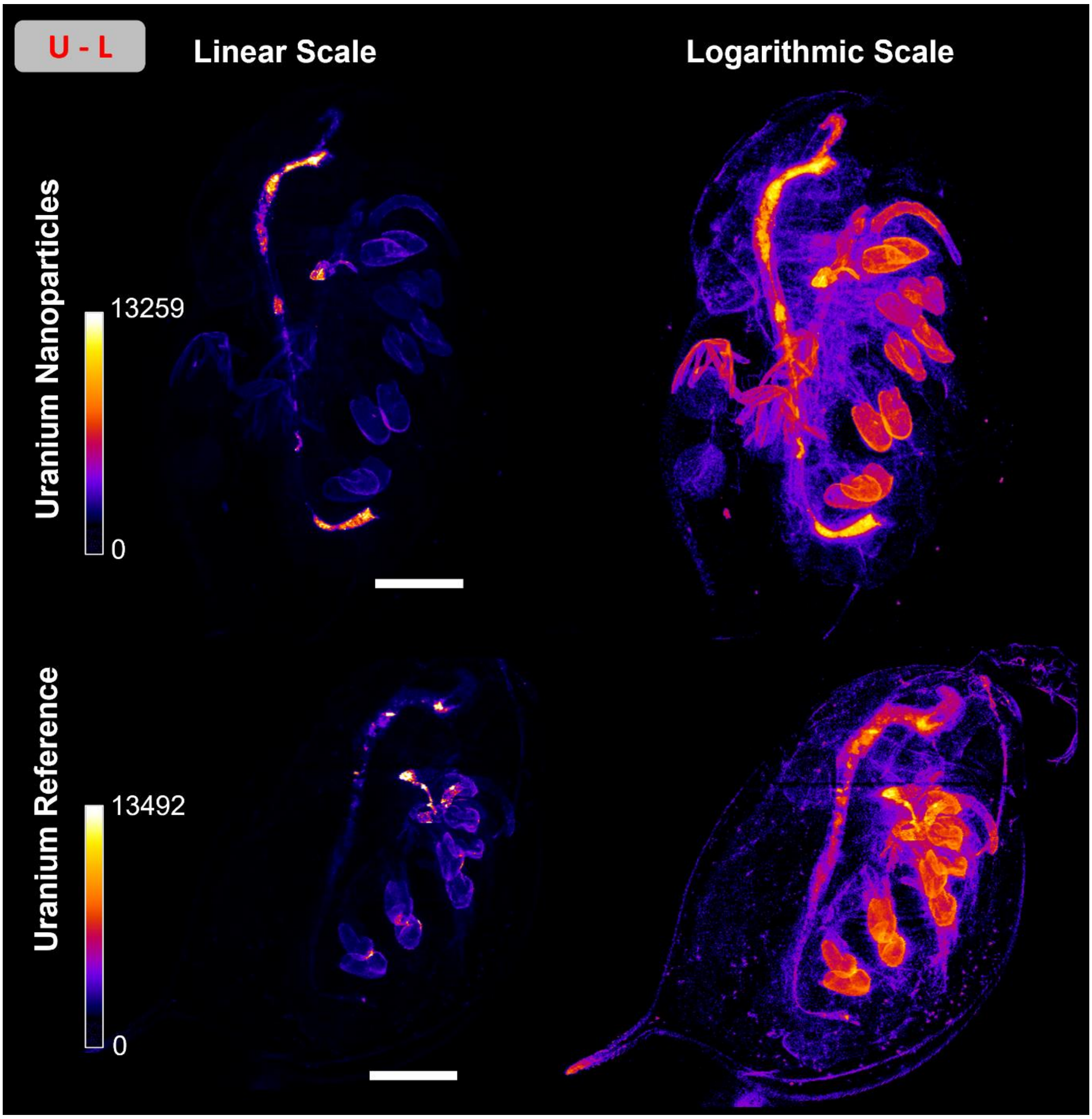

Figure S10: A comparison of U µ-XRF maps of UNP (*top*) and U_{Ref} (*bottom)* exposed daphnids shown in linear scale (*left*) and logarithmic scale (*right)*. Both measurements were conducted with a 5 µm step size and a 200 ms dwell time. All scalebars are 500 µm. Intensity scales show counts per second.

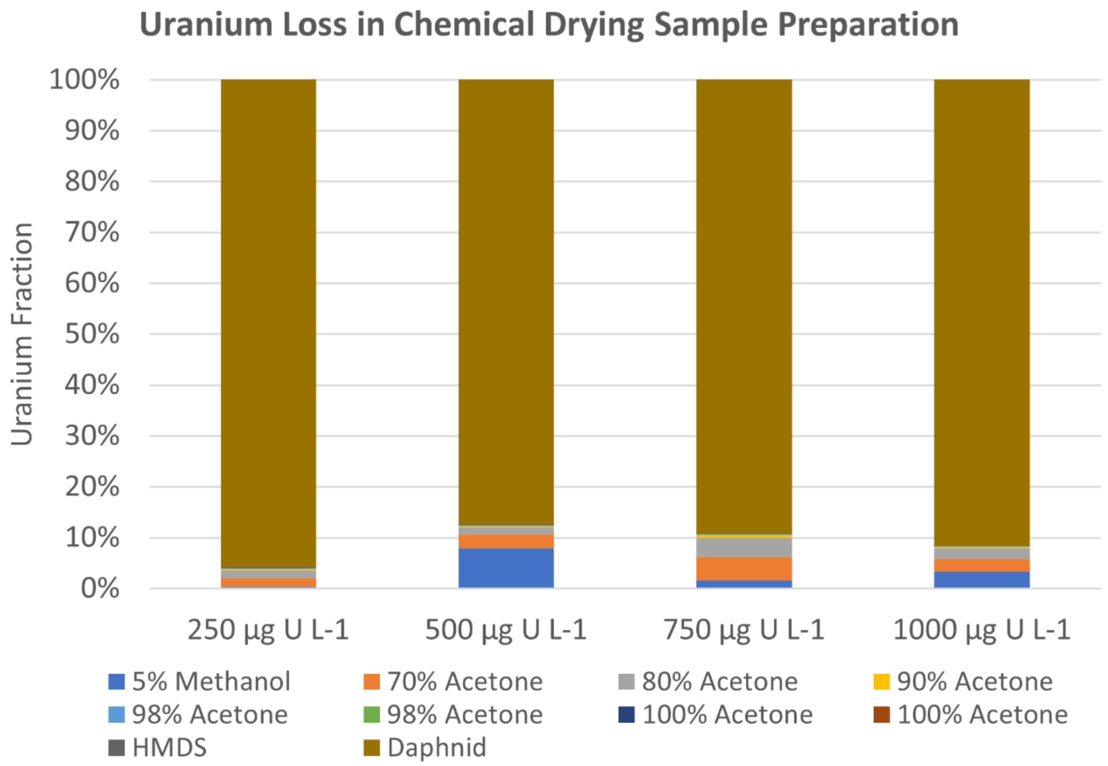

Figure S11: Loss of U from U exposed daphnids following chemical drying using acetone and HMDS. The fraction of U (in %) lost at each step of the sample preparation and in the daphnid were calculated from the amount of U (in ng) determined in each of the 9 solutions applied to daphnids from exposure experiments containing different U concentrations. Solutions were analyzed for total U concentrations were determined by ICP-QQQ.

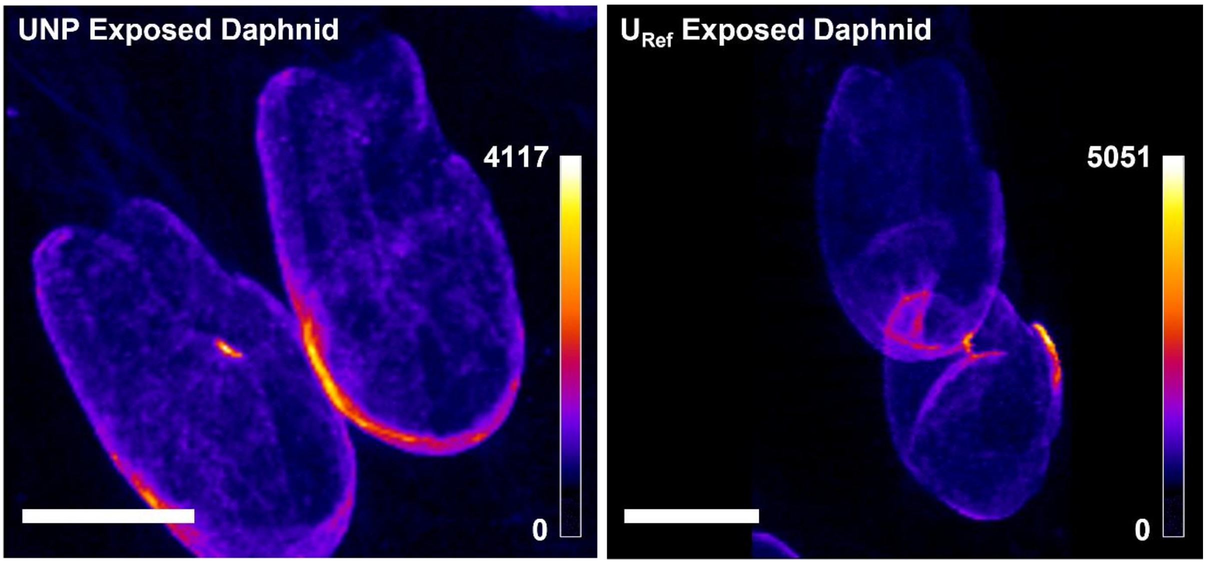

Figure S12: Uranium distribution around the lower middle pair of epipodites of the UNP exposed daphnid (*left*) and the U_{Ref} exposed daphnid (*right*). Both measurements were conducted with a 2 µm step size and a 200 ms dwell time. Intensity scales show counts per second and scale bars are 100 µm.

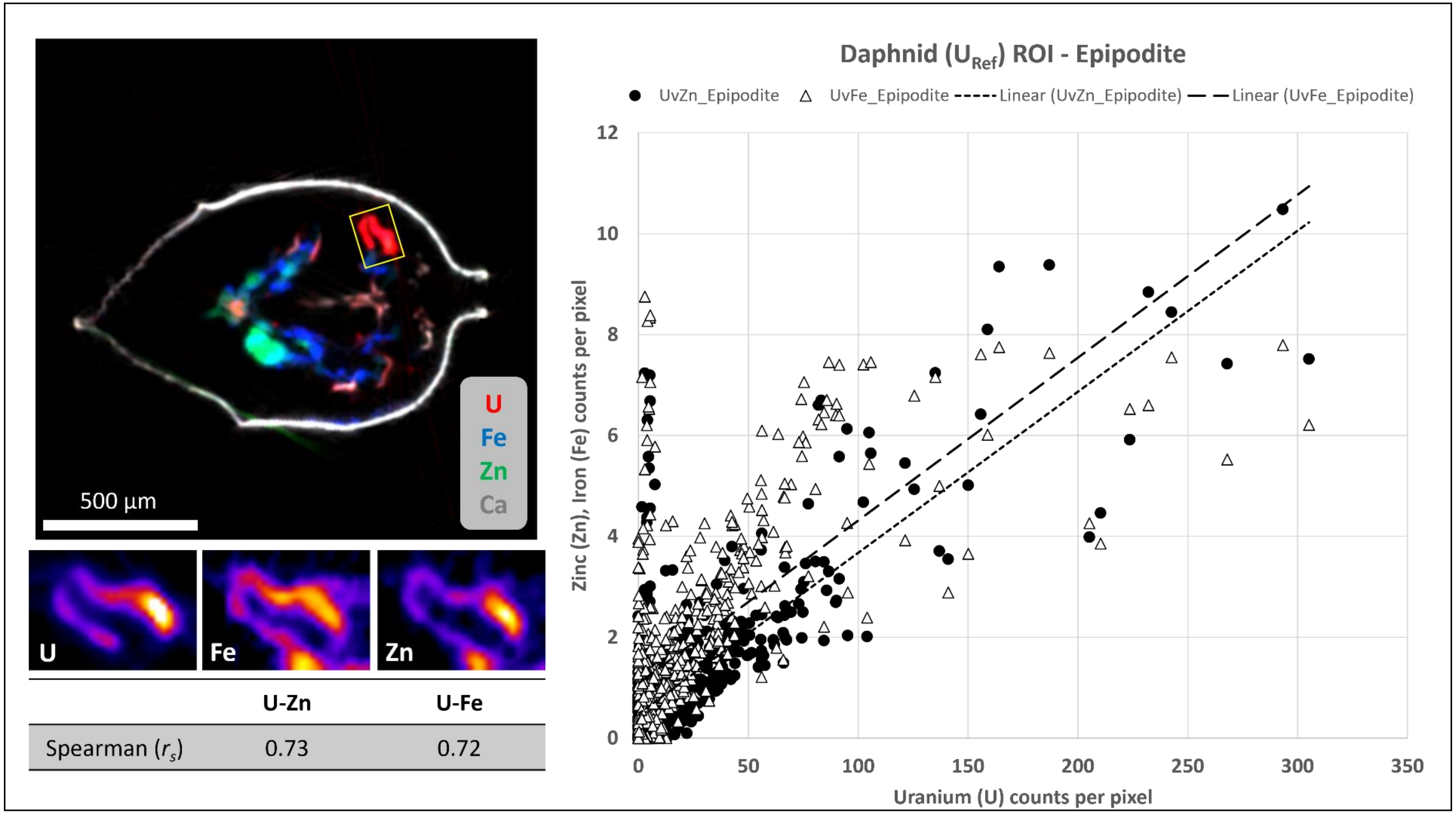

Figure S13: Correlation analysis of the U and Zn/Fe fluorescence signal collected on the epipodite featured in Figure 2B (U_{Ref} exposed daphnid). Within the tomographic section (*top left*), the epipodite is boxed in yellow. Counts per second of U, Fe and Zn for each pixel within this area (*bottom left*) were plotted as U vs. Zn and Fe (*right*). Spearman correlations for U-Zn (*p*-value = 3.7×10^{-87}) and U-Fe (*p*-value = 1.1×10^{-82}) showed statistically significant linear correlations.

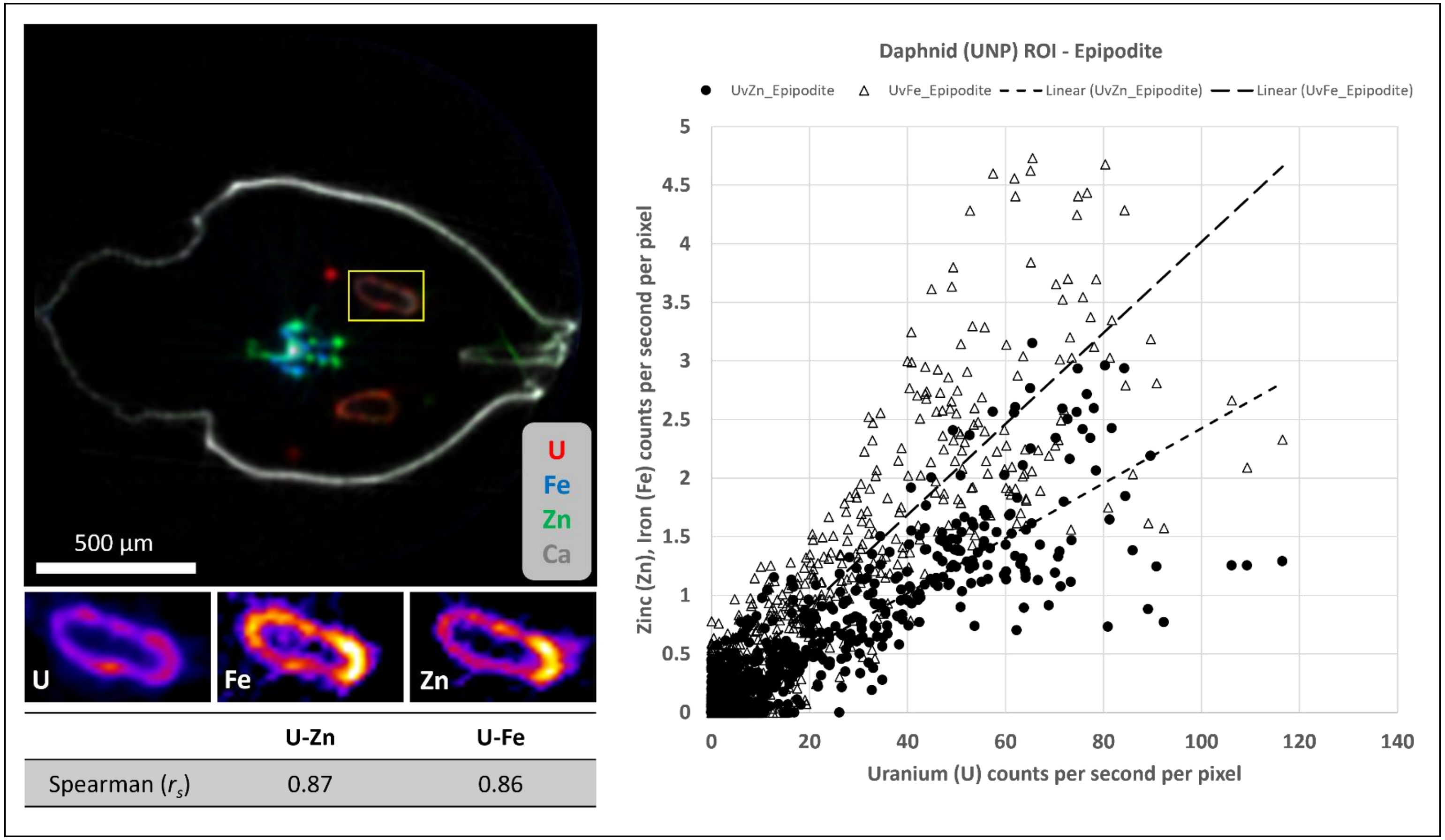

Figure S14: Correlation analysis of the fluorescence U and Fe/Zn signal collected on an epipodite from a UNP exposed daphnid. Within the tomopgraphic section (*top left*), the epipodite is boxed in yellow. Counts per second of U, Fe and Zn for each pixel within this area (*bottom left*) were plotted as U vs. Zn and Fe (*right*). Spearman correlations for U-Zn (p-value = 3.2×10^{-211}) and U-Fe (p-value = 1.2×10^{-202}) showed statistically significant linear correlations.

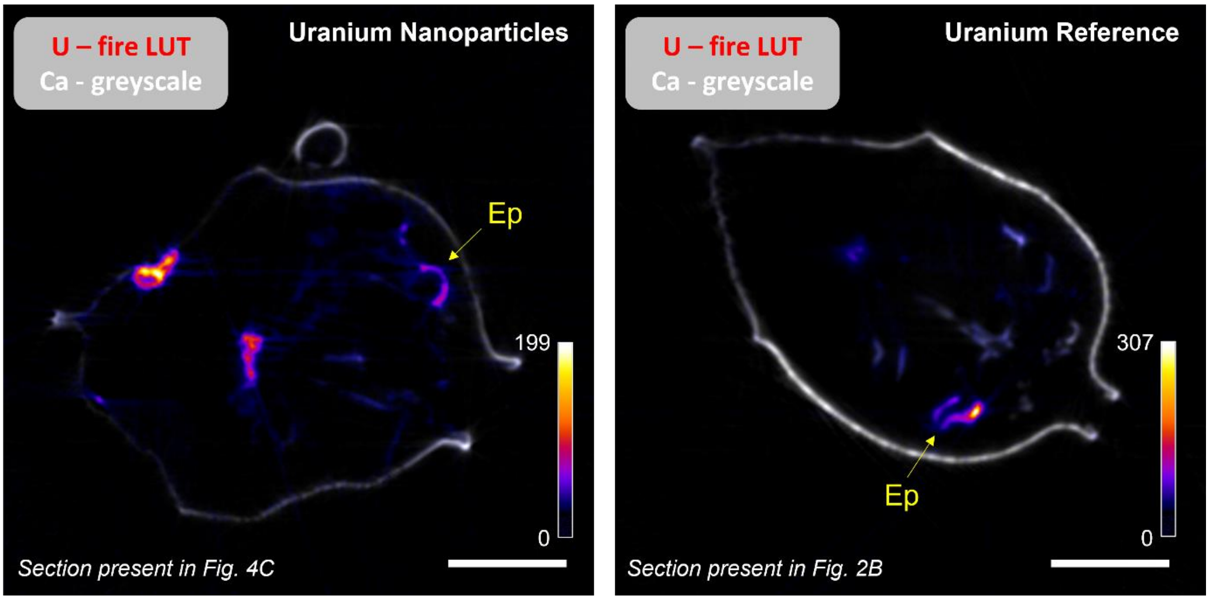

Figure S15: A comparison of tomographic sections displaying the dorsal U and Ca distributions on a UNP exposed daphnid (*left*) and a U_{Ref} exposed daphnid (*right*). The U distribution is displayed using the Fire LUT (ImageJ), while the Ca distribution is shown in grey scale to show the orientation of the section. The epipodite (Ep) is indicated with yellow arrows. All scalebars are 500 µm and intensity scales show counts per second.

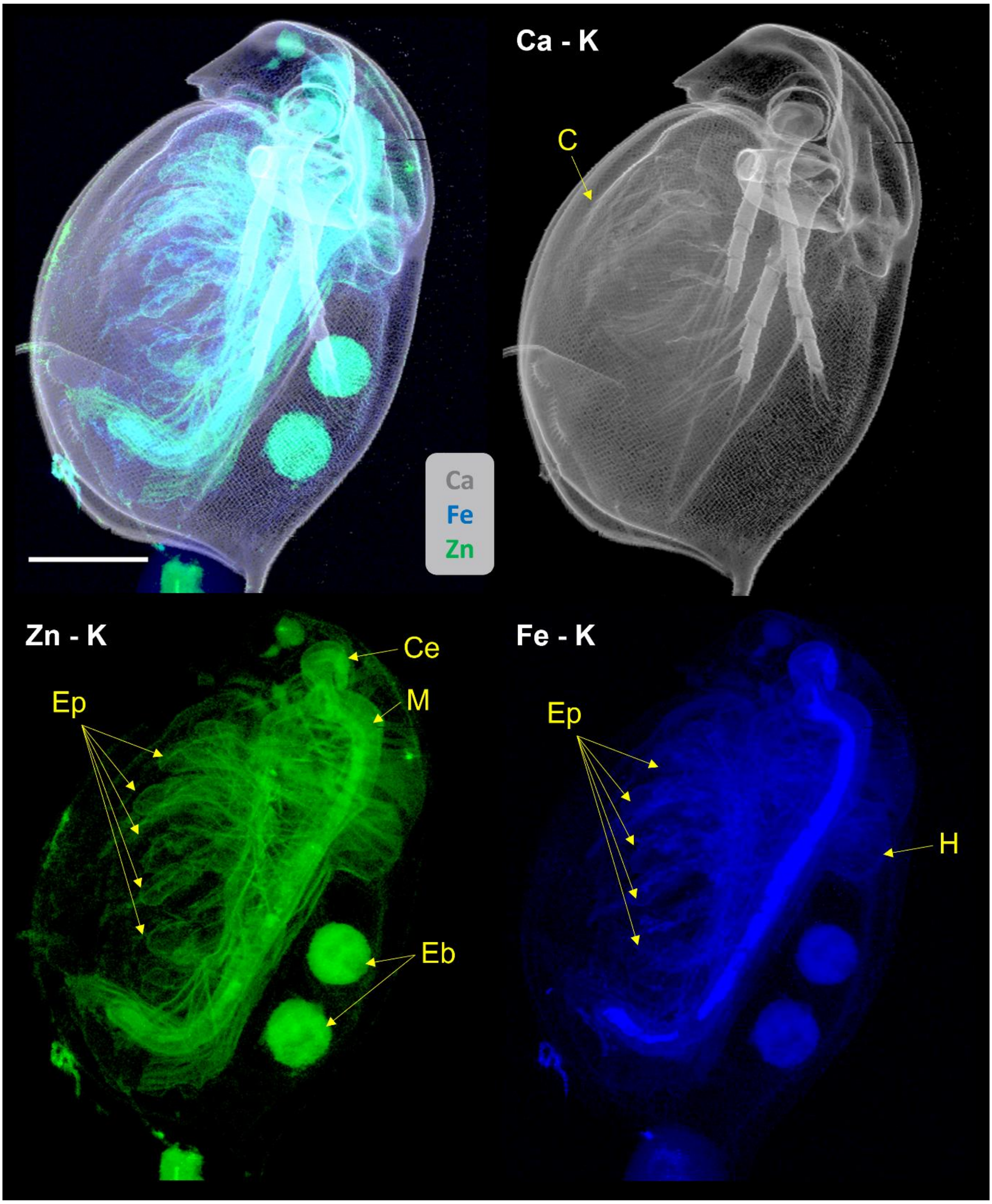

Figure S16: Combined (*top left*) and individual µ-XRF elemental mapping (Ca, Fe, Zn) of an unexposed, control daphnia (5 µm step size, 200 ms dwell time). All scale bars represent 500 µm and all signal intensities are scaled logarithmically. *Abbreviations:* carapace (C), hepatic ceca (Ce), midgut (M), epipodites (Ep), heart (H), and embryos (Eb).

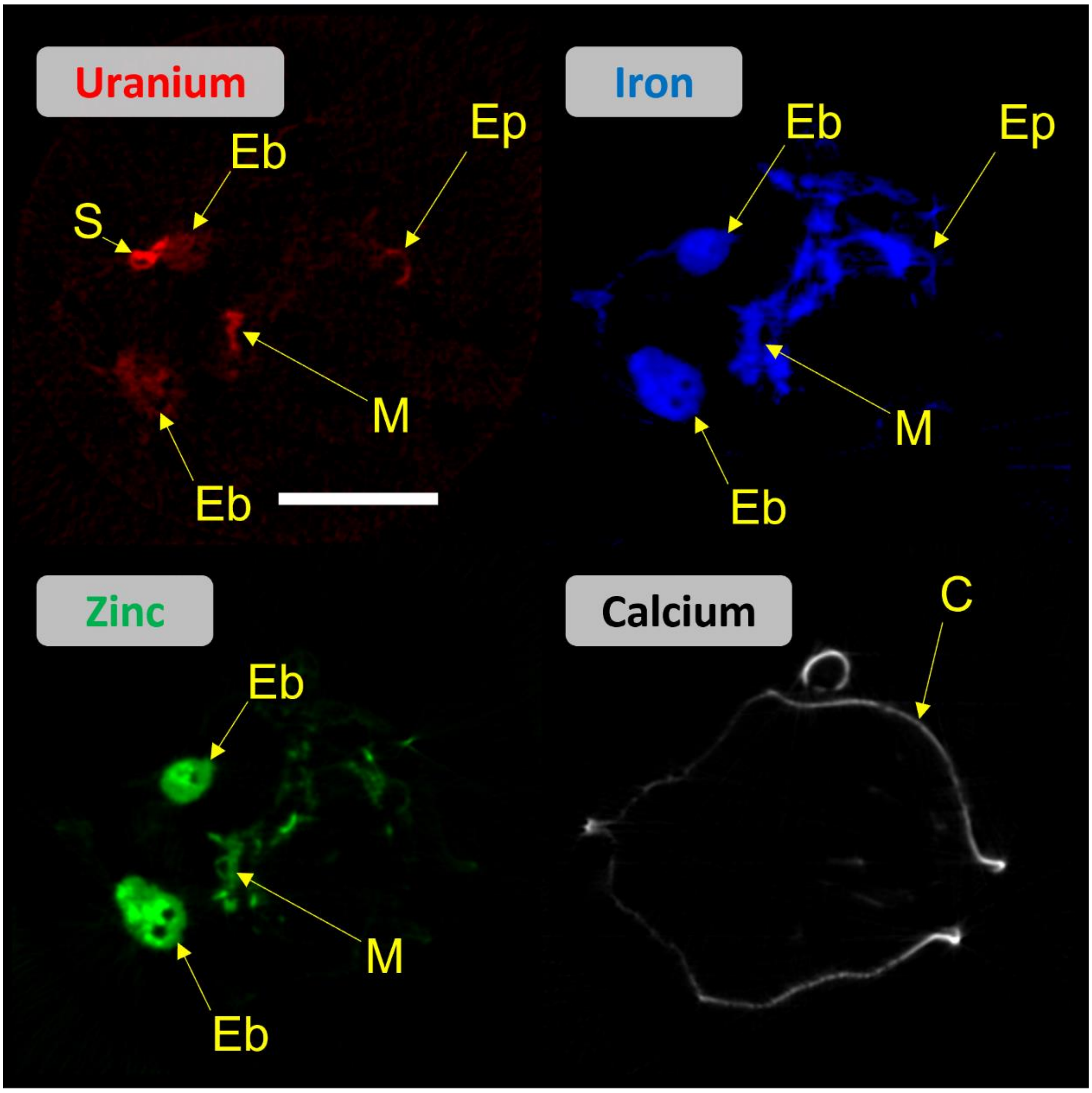

Figure S17: Individual elemental maps (U, Fe, Zn, and Ca) presented in the tomographic section featured in Figure 4C. Elemental signal intensities are in logarithmic scale. The scale bar represents 500 µm. *Abbreviations:* embryo (Eb), chorion structures (S), midgut (M), epipodite (Ep), and carapace (C).

Table S4: Reproduction test results for UNP and U_{Ref} exposure experiments.

**Uranium Nanoparticles**		**Uranium Reference Solution Exposures**	
Parent Exposure Concentration (ug U L^{-1})	Parent Clutch Size	Parent Exposure Concentration (ug U L^{-1})	Parent Clutch Size
8.8 ± 1.9	2	0 (A)	2
8.8 ± 1.9	1	0 (B)	4
8.8 ± 1.9	3	0 (C)	3
82.5 ± 12.7	2	10.8 ± 1.6	4
82.5 ± 12.7	3	10.8 ± 1.6	5
112 ± 4	3	10.8 ± 1.6	2
112 ± 4	3	91.4 ± 12.9	3
112 ± 4	2	91.4 ± 12.9	4
161 ± 12	2	91.4 ± 12.9	1
161 ± 12	5	139 ± 15	5
161 ± 12	4	139 ± 15	3
195 ± 13	2	139 ± 15	3
195 ± 13	3	203 ± 7	1
388 ± 10	1		

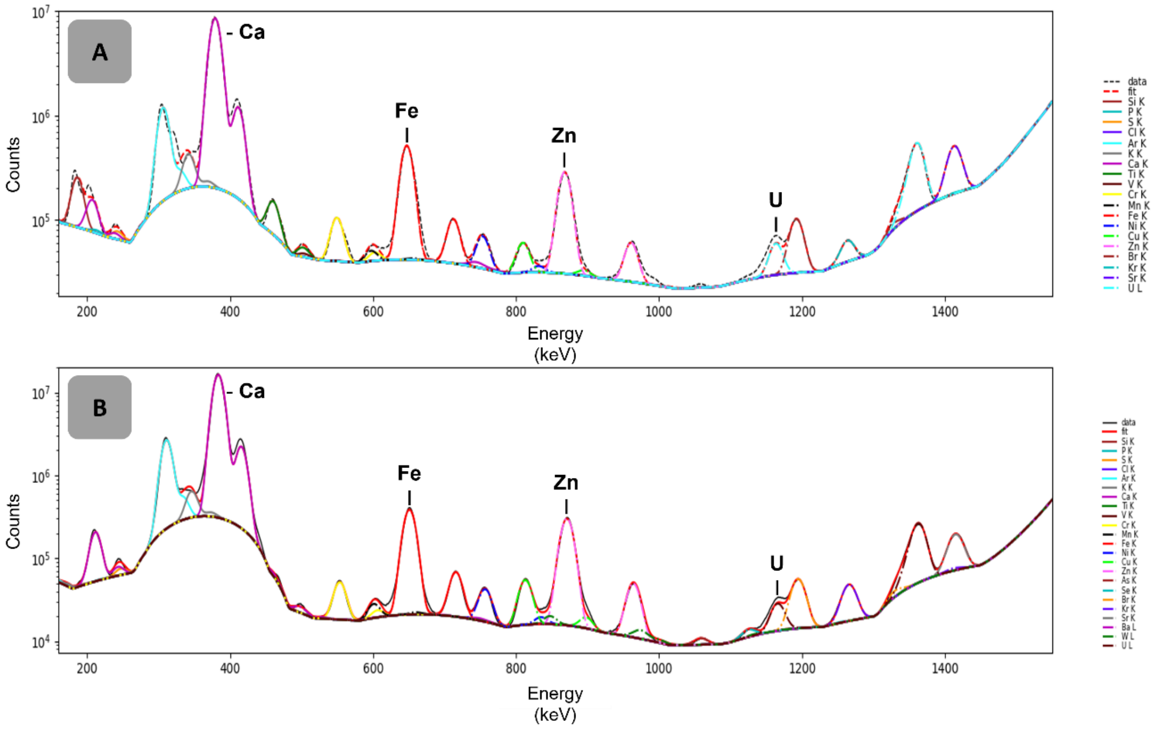

Figure S18: Sum XRF spectra of representative maps collected on the UNP (A) and U_{Ref} (B) exposed *Daphnia magna.* Element labels indicate the main energy peaks for Ca, Fe, Zn, and U.
